# Bone-targeted lncRNA OGRU alleviates unloading-induced bone loss via miR-320-3p/Hoxa10 axis

**DOI:** 10.1038/s41419-020-2574-1

**Published:** 2020-05-19

**Authors:** Ke Wang, Yixuan Wang, Zebing Hu, Lijun Zhang, Gaozhi Li, Lei Dang, Yingjun Tan, Xinsheng Cao, Fei Shi, Shu Zhang, Ge Zhang

**Affiliations:** 1 The Key Laboratory of Aerospace Medicine, Ministry of Education, Air Force Medical University, 710032 Xi’an, Shaanxi China; 2 Institute for Advancing Translational Medicine in Bone & Joint Diseases, School of Chinese Medicine, Hong Kong Baptist University, Hong Kong SAR, China; 3 State Key Laboratory of Space Medicine Fundamentals and Application, China Astronaut Research and Training Center, Beijing, 100094 China

**Keywords:** Mechanisms of disease, Long non-coding RNAs

## Abstract

Unloading-induced bone loss is a threat to human health and can eventually result in osteoporotic fractures. Although the underlying molecular mechanism of unloading-induced bone loss has been broadly elucidated, the pathophysiological role of long noncoding RNAs (lncRNAs) in this process is unknown. Here, we identified a novel lncRNA, OGRU, a 1816-nucleotide transcript with significantly decreased levels in bone specimens from hindlimb-unloaded mice and in MC3T3-E1 cells under clinorotation-unloading conditions. OGRU overexpression promoted osteoblast activity and matrix mineralization under normal loading conditions, and attenuated the suppression of MC3T3-E1 cell differentiation induced by clinorotation unloading. Furthermore, this study found that supplementation of pcDNA3.1(+)–OGRU via (DSS)_6_–liposome delivery to the bone-formation surfaces of hindlimb-unloaded (HLU) mice partially alleviated unloading-induced bone loss. Mechanistic investigations demonstrated that OGRU functions as a competing endogenous RNA (ceRNA) to facilitate the protein expression of Hoxa10 by competitively binding miR-320-3p and subsequently promote osteoblast differentiation and bone formation. Taken together, the results of our study provide the first clarification of the role of lncRNA OGRU in unloading-induced bone loss through the miR-320-3p/Hoxa10 axis, suggesting an efficient anabolic strategy for osteoporosis treatment.

## Introduction

Bone remodeling is a complex process orchestrated by the dynamic balance between osteoblast-mediated bone formation and osteoclast-mediated bone resorption^1^. This balance can be regulated by a variety of factors, such as hormones, cytokines, and mechanical stimuli^2^. The effect of mechanical stimuli on bone remodeling has been described by Wolff’s law: the structure and function of bone are adapted to its mechanical environment^3^. Accumulating evidence has shown that prolonged mechanical unloading by long-duration space flight and extended bed rest can induce disuse osteoporosis in humans^4,5^. The most devastating consequence of osteoporosis is fractures, which are associated with an enormous burden of health care costs, morbidity, and mortality. Existing antiosteoporotic drugs can effectively reduce the incidence of fractures in patients with osteoporosis. However, the major side effects of these drugs, such as osteonecrosis, hypercalcemia, and thromboembolic diseases, can be extremely harmful to human health. Therefore, it is necessary to further explore the molecular mechanism and develop more effective measures to counteract unloading-induced bone loss^6^.

HLU mice, an established model used to simulate skeletal unloading, show irreversible bone loss via the inhibition of bone formation, and the promotion of bone resorption^5,7–9^. In vitro experiments in ground-based facilities for simulating microgravity unloading, such as 2D clinostat, random positioning machine (RPM), rotating wall vessel (RWV) bioreactor, and magnetic levitator, indicated that the dysfunction of osteoblasts caused by unloading can disturb their ability to translate mechanical loading into biochemical signals, which can partly explain the occurrence of unloading-induced bone loss^10–13^, and many intracellular signaling pathways have been reported to participate in this complex process^5,8,14^. To further understand the pathophysiology of unloading-induced bone loss and identify new biological targets, we selected 2D clinorotation and HLU models for simulating unloading conditions in vitro and in vivo.

Whole-genome sequencing results show that ~93% of the sequences in the human genome can be transcribed, among which no more than 2% have protein-coding ability; 98% of the transcriptome consists of noncoding RNAs without protein-coding potential^15^. Long noncoding RNA are transcripts of more than 200 nucleotides in length, without long open-reading frames, but often with mRNA structural features (a 5′ cap and a polyA tail)^16^. Previous studies have found that lncRNAs can participate in a wide range of biological processes by regulating gene expression at the transcriptional, post-transcriptional, and epigenetic levels^17^. For example, lncRNAs can induce a repressive chromatin state by recruiting chromatin-modifying complexes, such as PRC2, Mll, PcG, and G9a methyltransferase, to specific genomic loci^18–22^. Furthermore, lncRNAs can recruit and modulate the activity of the RNA-binding protein TLS, and subsequently cause the repression of cyclin D1 transcription in human cell lines^23^. Moreover, many cytoplasmic lncRNAs can competitively bind to microRNAs by functioning as competitive endogenous RNAs (ceRNAs) and thereby regulate protein translation^24–27^. Importantly, although the roles of lncRNAs in the process of osteogenesis have been widely reported^28–30^, only a few studies have shown that lncRNAs are involved in the occurrence and development of unloading-induced bone loss^31,32^.

In this study, we found that the lncRNA OGRU is downregulated during unloading, and upregulated during osteoblast differentiation. OGRU overexpression promoted osteoblast activity and matrix mineralization under both normal loading and unloading conditions in vitro. In an in vivo experiment, a (DSS)_6_–liposome delivery system was used to deliver pcDNA3.1(+)–OGRU specifically to bone-formation surfaces^33,34^. The results showed that supplementation with (DSS)_6_–liposome–OGRU can effectively promote osteoblastic bone formation, increase bone mass, and improve bone microarchitecture and biomechanical properties in HLU mice. In addition, OGRU facilitated the protein expression of Hoxa10 by competitively binding miR-320-3p, which in turn promoted osteoblast differentiation and bone formation. In summary, our study identified a new regulator of unloading-induced bone loss, and may establish a potential therapeutic strategy against osteoporosis.

## Results

### OGRU is a novel lncRNA sensitive to unloading and is upregulated during osteoblast differentiation of MC3T3-E1 cells

**Fig. 1:**
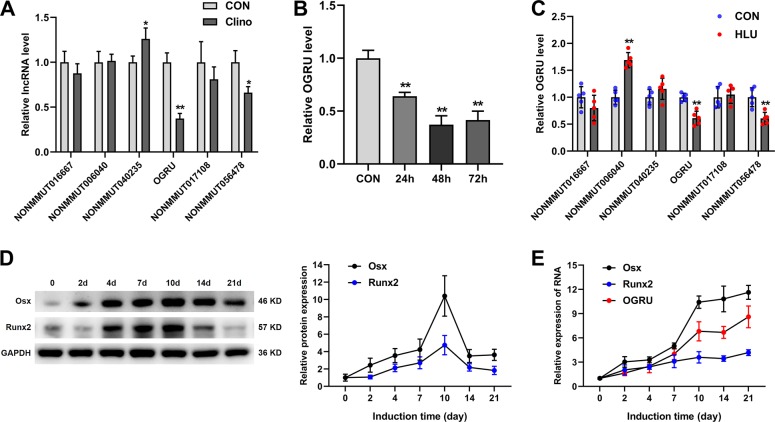
OGRU is sensitive to unloading and is upregulated during osteoblast differentiation of MC3T3-E1 cells. **a** Differential expression of lncRNAs selected from previous microarray assay data was determined by qRT-PCR in MC3T3-E1 cells under clinorotation-unloading condition for 48 h (*n* = 3). **b** qRT-PCR analysis of OGRU expression in MC3T3-E1 cells under clinorotation-unloading condition for 24, 48, and 72 h (*n* = 3). **c** qRT-PCR analysis of lncRNA expression in the tibias of control (CON) and HLU mice (*n* = 5). **d** The protein expression of Osx and Runx2 was determined by western blotting during osteoblastic differentiation of MC3T3-E1 cells (*n* = 3). **e** qRT-PCR analysis of Osx, Runx2, and OGRU RNA expression during osteoblast differentiation of MC3T3-E1 cells (*n* = 3). All data are the mean ± SD. *P < 0.05, **P < 0.01.

To identify unloading-sensitive lncRNAs, a microarray assay was performed in our previous work to detect differential expression of lncRNAs in MC3T3-E1 preosteoblast cells under clinorotation- unloading condition for 48 h^35^. The expression of candidate lncRNAs selected from the microarray assay data according to the fold change in expression was further validated by qRT-PCR. The results showed that NONMMUT068562 (lnc-OGRU) was markedly decreased in the clinorotation-unloading group, and that this change persisted for at least 72 h in MC3T3-E1 cells (Fig. 1a, b). In addition, NONMMUT068562 (lnc-OGRU) was also markedly downregulated in the tibias of HLU mice (Fig. 1c). To identify whether NONMMUT068562 (lnc-OGRU) is potentially involved in osteoblast differentiation, MC3T3-E1 cells were cultured in osteogenic medium for 21 days. We found that the expression of OGRU increased gradually, and was positively associated with the mRNA and protein expression of the osteogenic marker genes Osx and Runx2 during osteoblast differentiation (Fig. 1d, e). Thus, this uncharacterized lncRNA was selected for further study, and was named OGRU. Next, 5′ and 3′ rapid amplification of cDNA end (RACE) assays showed that OGRU was a 1816-nucleotide transcript comprising two exons and located on chromosome 9, and northern blot analysis further confirmed the expected size (Supplementary Fig. 1A, B). OGRU displayed no protein-coding potential, as predicted by the coding-potential calculator (CPC) and the coding-potential assessment tool (CPAT) (Supplementary Table 1). In summary, we identified a novel lncRNA, OGRU, which exhibited significantly decreased expression during unloading both in vitro and in vivo, and was associated with osteoblast differentiation of MC3T3-E1 cells.

### OGRU promotes the osteoblast activity and matrix mineralization and attenuates the osteogenic decline of MC3T3-E1 cells induced by clinorotation unloading

**Fig. 2:**
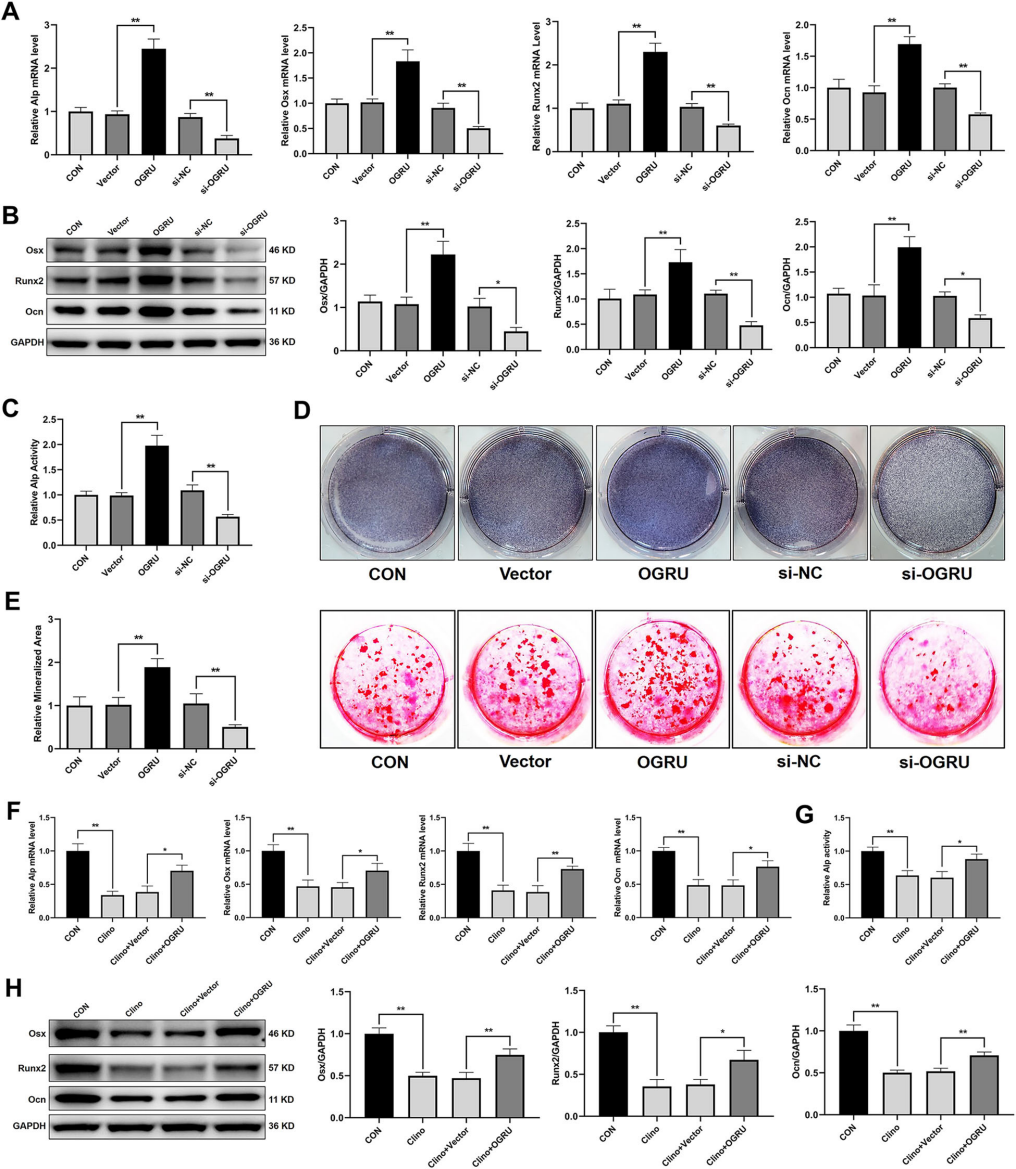
OGRU promotes the osteoblast activity and matrix mineralization, and attenuates the osteogenic decline of MC3T3-E1 cells induced by clinorotation unloading. MC3T3-E1 cells were transfected with pcDNA3.1(+)–OGRU, si-OGRU, or the corresponding controls, and were cultured in osteogenic medium. **a** The mRNA levels of Alp, Osx, Runx2, and Ocn were measured by qRT-PCR 4 days after osteogenic treatment (*n* = 3). **b** The protein levels of Osx, Runx2, and Ocn were measured by western blotting 4 days after osteogenic treatment (*n* = 3). **c** The relative Alp activity, 4 days after osteogenic treatment (*n* = 3). **d** Representative images of Alp staining in MC3T3-E1 cells 7 days after osteogenic treatment (*n* = 3). **e** Representative images of alizarin red staining in MC3T3-E1 cells 21 days after osteogenic treatment are shown, and relative areas of mineralization were quantified by Image J (*n* = 3). MC3T3-E1 cells were transfected with pcDNA3.1(+) or pcDNA3.1(+)–OGRU and cultured in clinorotation-unloading condition for 48 h, and were then subjected to qRT-PCR analysis of Alp, Osx, Runx2, and Ocn mRNA levels (**f**), measurement of the relative Alp activity (**g**), and western blot analysis of Osx, Runx2, and Ocn protein expression (**h**) (*n* = 3). All data are the mean ± SD. *P < 0.05, **P < 0.01.

To assess the function of OGRU during osteoblast differentiation, MC3T3-E1 cells were transfected with pcDNA3.1(+)–OGRU, si-OGRU, or the corresponding controls, and were cultured in the osteogenic medium. The results showed that transient transfection generates nonphysiological amounts of OGRU (Supplementary Fig. 2). Notably, overexpression of OGRU in MC3T3-E1 cells significantly increased the mRNA levels of osteogenic marker genes, including Alp, Osx, Runx2, and Ocn (Fig. 2a). Consistent with this finding, the protein expression levels of Osx, Runx2, Ocn, and Alp activity were increased in OGRU-overexpressing MC3T3-E1 cells (Fig. 2b, c). Alp staining was significantly enhanced in the group treated with pcDNA3.1(+)–OGRU (Fig. 2d). In addition, matrix mineralization, as visualized by alizarin red staining, was significantly promoted by pcDNA3.1(+)–OGRU (Fig. 2e). In contrast, MC3T3-E1 cells transfected with si-OGRU exhibited impaired osteoblast activity and matrix mineralization. These data suggested that OGRU is a critical regulator of osteoblast activity and matrix mineralization in vitro under normal conditions. To explore whether forced expression of OGRU could rescue the osteogenic decline induced by unloading in vitro, MC3T3-E1 cells were transfected with pcDNA3.1(+)–OGRU for 12 h, and were then cultured in clinorotation-unloading condition for 48 h. These results showed that OGRU substantially attenuated the reduction in the mRNA levels of osteogenic marker genes, including Alp, Osx, Runx2, and Ocn, induced by clinorotation unloading (Fig. 2f). Consistent with this result, the protein expression levels of Osx, Runx2, Ocn, and Alp activity were also attenuated in OGRU-overexpressing MC3T3-E1 cells (Fig. 2g, h).

### Assessment of the targeting ability of (DSS)_6_–liposome delivery system to bone-formation surfaces and toxicity in vivo

**Fig. 3:**
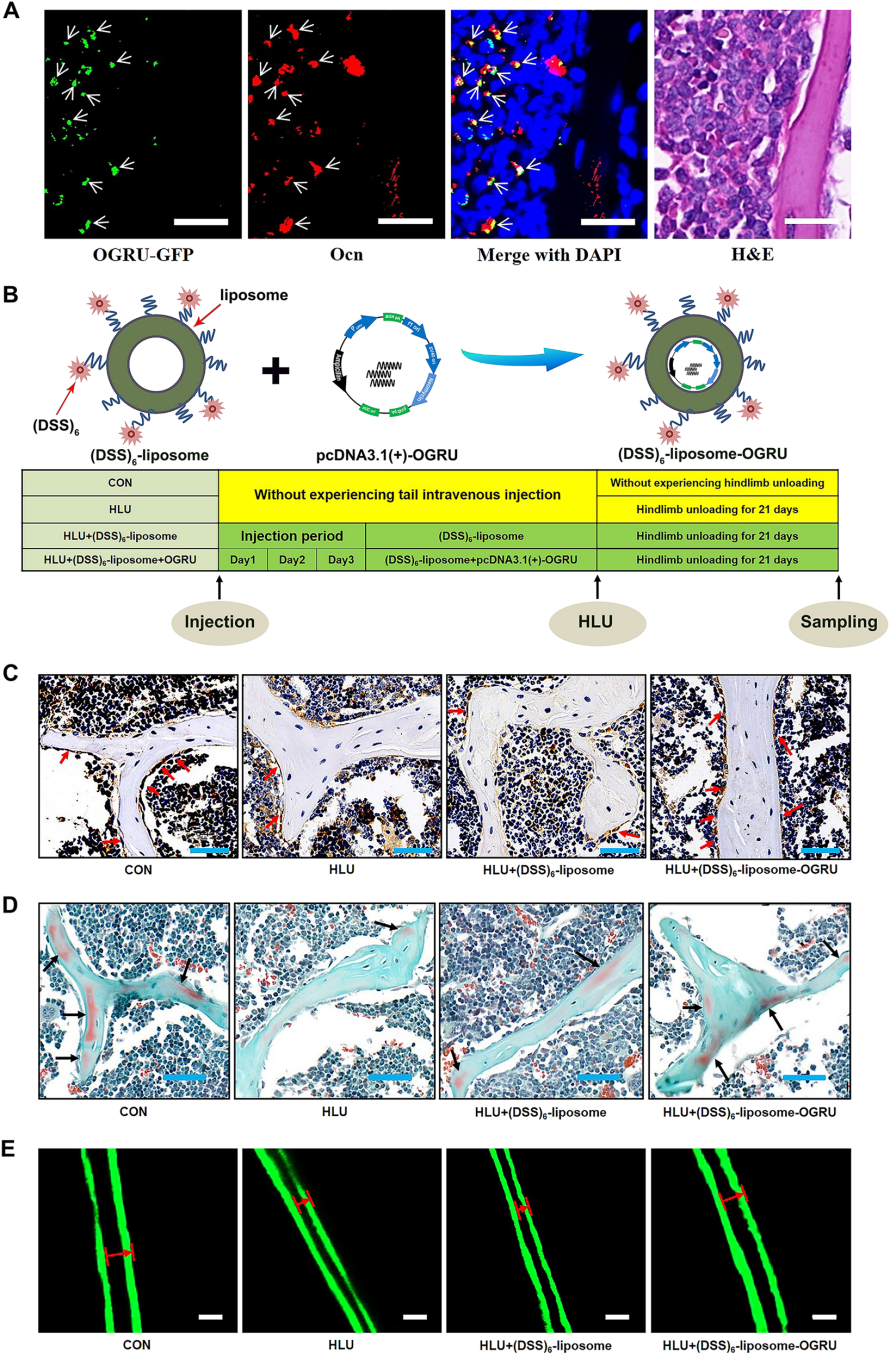
The effect of bone-targeted OGRU on the function of osteoblasts in HLU mice. **a** Representative images of immunofluorescence staining and H&E staining of same sections in the femurs of mice. The pcDNA3.1(+)–OGRU was labeled with GFP (green, left column). Immunofluorescence staining of Ocn was performed to detect osteoblasts (red, middle-left column). Merged images with DAPI staining showed co-staining of pcDNA3.1(+)–OGRU and osteoblasts. H&E staining was included in the right column. Scale bar = 20 μm (*n* = 5). **b** Schematic of the experimental design. **c** Representative images of osteocalcin immunohistochemical staining in the femurs of mice in the indicated groups (*n* = 5). Scale bars: 50 μm. **d** Representative images of Goldner’s trichrome staining of the trabecular bone at distal femurs of mice in the indicated groups (*n* = 5). Osteoid stains are shown in red (indicated by the black arrows), and mineralized bone stains in green. Scale bars: 50 µm. **e** Representative images of calcein double labeling (*n* = 5). Scale bars: 10 μm. All data are the mean ± SD. *P < 0.05, **P < 0.01. NS not significant.

As we found that OGRU played an important role in osteoblast activity and matrix mineralization under both normal and unloading conditions in vitro, we further explored whether therapeutic overexpression of OGRU could rescue unloading-induced bone loss in HLU mice. To evaluate the efficacy and safety of (DSS)_6_–liposome delivery system, the organs of mice were collected for analysis 3 days after a single injection of (DSS)_6_–liposome–OGRU–GFP or PBS. The qRT-PCR analysis showed that bone-targeted OGRU effectively increased the expression of OGRU in bone, but failed to affect the expression of OGRU in the other organs (Supplementary Fig. 3). Besides, representative immunofluorescence staining micrographs showed co-staining of OGRU–GFP and Ocn-positive cells (osteoblasts) at bone-formation surfaces in the bones of (DSS)_6_–liposome–OGRU–GFP-treated mice (Fig. 3a). Importantly, the organs of mice treated with (DSS)_6_–liposome–OGRU–GFP showed no obvious pathological abnormalities and tissue damage, which proved that (DSS)_6_–liposome–OGRU do not cause significant toxic effects in vivo (Supplementary Fig. 4).

### Bone-targeted OGRU promotes the function of osteoblasts in HLU mice

To explore the protective effect of bone-targeted OGRU on unloading-induced bone loss in HLU mice, we preinjected pcDNA3.1(+)–OGRU carried by (DSS)_6_–liposome three times before HLU (Fig. 3b). After 21 days of HLU, mice were sacrificed, and the bilateral femurs and tibias were collected for bone analysis. The qRT-PCR results showed that the expression of OGRU in bone increased in bone-targeted OGRU group compared with the CON group, which partially counteracted the decreased mRNA expression of osteogenic markers (Alp, Osx, Runx2, and Ocn) in HLU mice (Supplementary Fig. 5). In addition, more Ocn-positive cells (osteoblasts) were found in the distal femurs from (DSS)_6_–liposome–OGRU-treated mice than in the femurs of (DSS)_6_–liposome-treated mice, as indicated by immunohistochemical staining (Fig. 3c). To evaluate the role of bone-targeted OGRU in new bone formation in HLU mice, Goldner’s trichrome staining was performed and showed more newly formed bone in the distal femurs from (DSS)_6_–liposome–OGRU-treated mice than in the distal femurs from (DSS)_6_–liposome-treated mice (Fig. 3d). Moreover, calcein double-labeling analysis showed a higher mineral apposition rate (MAR) in the (DSS)_6_–liposome–OGRU group than in the (DSS)_6_–liposome group (Fig. 3e; Supplementary Fig. 6).

### Bone-targeted OGRU improves bone phenotypes in HLU mice

**Fig. 4:**
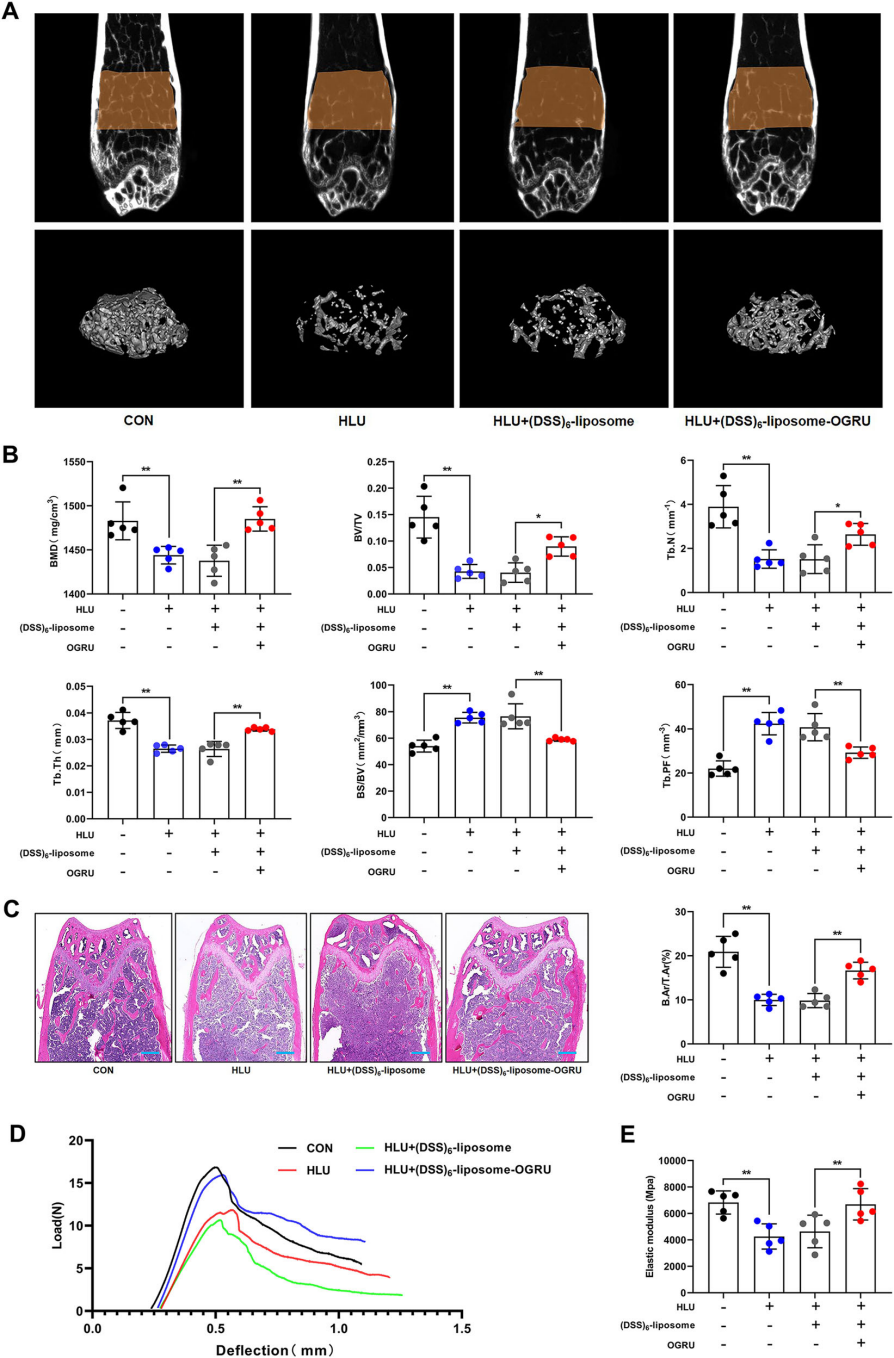
Bone-targeted OGRU improves trabecular bone phenotypes in HLU mice. **a** Representative images of micro-CT 2D sections and 3D reconstruction of distal femurs of mice in the indicated groups (*n* = 5). **b** Micro-CT measurement of bone mineral density (BMD), the ratio of bone volume to total volume (BV/TV), the ratio of bone surface to bone volume (BS/BV), trabecular bone number (Tb.N), trabecular thickness (Tb.Th), and trabecular bone pattern factor (Tb.PF) (*n* = 5). **c** H&E staining showing trabecular microarchitecture and quantitative analysis of the bone area/total area on the proximal side of the growth plate in the distal femurs of mice in the indicated groups (*n* = 5). Scale bars: 300 μm. **d** Representative load-deflection curves for the respective groups (*n* = 5). **e** The elastic modulus was calculated (*n* = 5). All data are the mean ± SD. *P < 0.05, **P < 0.01.

Next, we further explored the effects of bone-targeted OGRU on bone phenotypes. Micro-CT assays revealed that the significant decreases in bone mineral density (BMD), the ratio of bone volume to total volume (BV/TV), trabecular bone number (Tb.N), trabecular thickness (Tb.Th) and cortical thickness (Ct.Th), and the increases in the ratio of bone surface to bone volume (BS/BV) and trabecular bone pattern factor (Tb.PF) induced by HLU were efficiently attenuated in the (DSS)_6_–liposome–OGRU group (Fig. 4a, b; Supplementary Fig. 7). H&E staining of the proximal side of the growth plate in the distal femurs further confirmed that (DSS)_6_–liposome–OGRU counteracted the low-bone-mass phenotype of HLU mice, as quantified by the ratio of bone area to total area (B.Ar/T.Ar) (Fig. 4c). These data demonstrated that the low bone mass and deterioration of the trabecular microarchitecture in HLU mice were alleviated by bone-targeted OGRU. Biomechanical properties can indicate the resistance of bones to fracture, so we performed a three-point bending test on femurs to evaluate bone stiffness and strength. The structural parameters of the femurs, including the maximum load, stiffness, and elastic modulus, were calculated based on the load-deflection curves. The results showed that these three parameters were substantially decreased by HLU, but the impairment of these biomechanical properties was significantly ameliorated by (DSS)_6_–liposome–OGRU (Fig. 4d, e; Supplementary Fig. 8).

### OGRU interacts with miR-320-3p

**Fig. 5:**
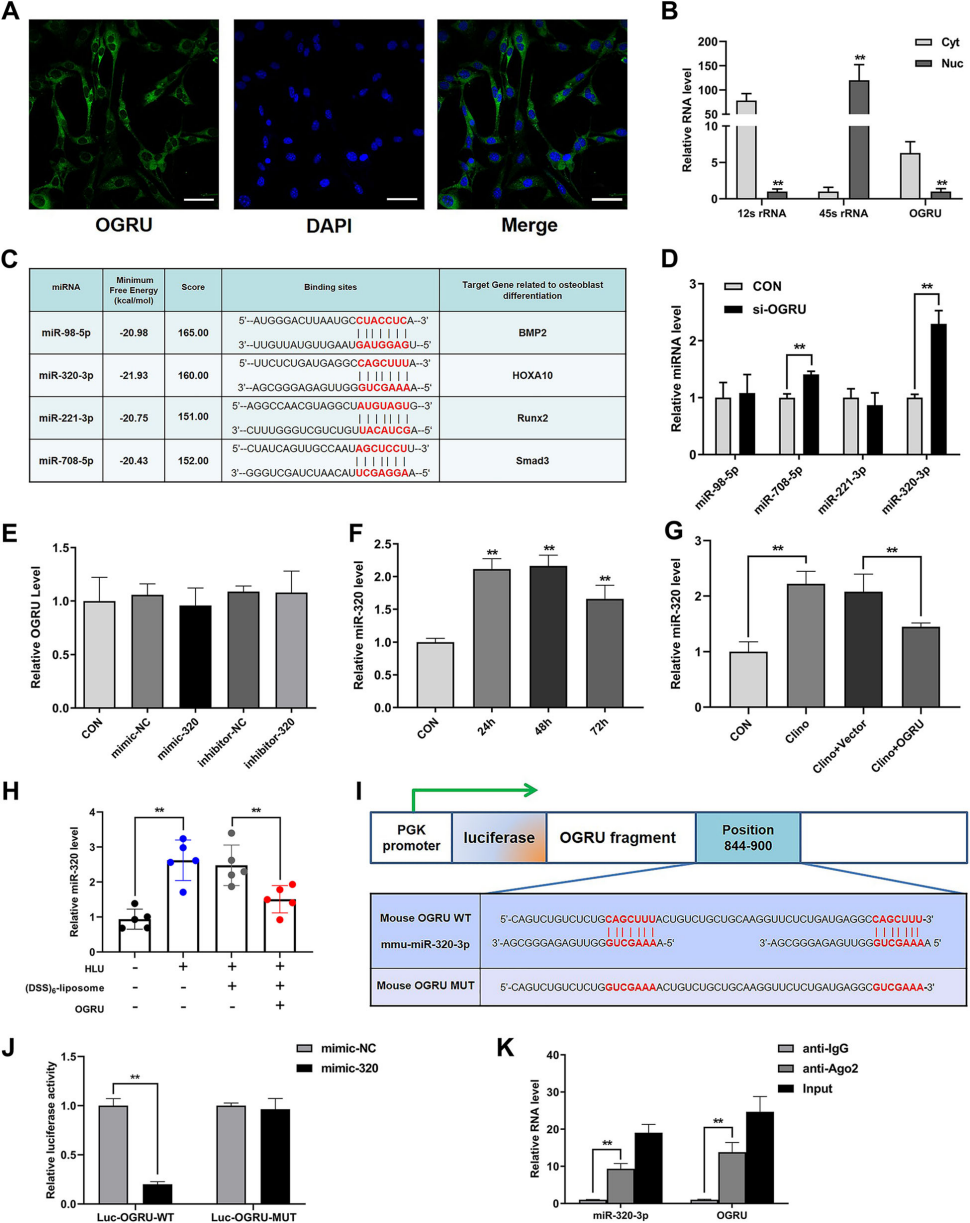
OGRU interacts with miR-320-3p. **a** RNA fluorescence in situ hybridization showed that OGRU primarily localizes in the cytoplasm (*n* = 3). Scale bars: 50 µm. **b** qRT-PCR analysis of OGRU expression in the cytoplasmic (Cyt) and nuclear (Nuc) fractions. The control, 45S ribosomal RNA (rRNA), was primarily localized in the nucleus, and 12S rRNA was found primarily in the cytoplasmic fraction (*n* = 3). **c** The RegRNA2.0 prediction for the binding sites for candidate miRNAs in the OGRU transcript. **d** qRT-PCR analysis of miRNA levels after treatment with si-OGRU in MC3T3-E1 cells (*n* = 3). **e** qRT-PCR analysis of OGRU expression levels after treatment with mimic-320, inhibitor-320, or the corresponding controls (*n* = 3). **f** qRT-PCR measurement of the relative miR-320-3p levels in MC3T3-E1 cells under clinorotation-unloading condition for 24, 48, and 72 h (*n* = 3). **g** qRT-PCR measurement of miR-320-3p levels after MC3T3-E1 cells were transfected with pcDNA3.1(+)–OGRU and subjected to clinorotation unloading for 48 h (*n* = 3). **h** qRT-PCR detection of the miR-320-3p levels in the tibias from mice in the CON, HLU, HLU + (DSS)_6_–liposome and HLU + (DSS)_6_–liposome–OGRU groups (*n* = 5). **i**, **j** The relative luciferase activity of luciferase reporters containing OGRU wild-type (WT) or mutated (MUT) miR-320-binding sites in 293T cells cotransfected with mimic-320 or its negative control was measured. The data are presented as the relative ratio of firefly luciferase activity to renilla luciferase activity (*n* = 3). **k** Anti-AGO2 RIP was performed using input from cell lysate, normal mouse IgG, or anti-Ago2, followed by qRT-PCR to measure the OGRU and miR-320 levels (*n* = 3). All data are the mean ± SD. *P < 0.05, **P < 0.01.

As the function of OGRU on osteogenesis during unloading was confirmed both in vitro and in vivo, we further explored the mechanisms involved in this process. The mechanism of lncRNA is closely related to its subcellular location, which was examined by RNA fluorescence in situ hybridization and cell fractionation followed by qRT-PCR, and the results showed that OGRU primarily localizes in the cytoplasm (Fig. 5a, b). Thus, we speculated that OGRU may act as a ceRNA that binds to microRNAs. Bioinformatics analysis by RegRNA2.0 (http://regrna2.mbc.nctu.edu.tw/) revealed that the OGRU sequence contains several putative binding sites for microRNAs, and we selected four miRNAs involved in osteoblast differentiation among these putative binding miRNAs for further study^36–40^ (Fig. 5c; Supplementary Table 2). The results demonstrated that the level of miR-320-3p was markedly increased after treatment with si-OGRU, but miR-320-3p had no effect on the expression of OGRU (Fig. 5d, e). In addition, the miR-320-3p level was significantly increased during unloading in vitro and in vivo, and this effect was alleviated by OGRU overexpression (Fig. 5f–h). Next, the binding between miR-320-3p and OGRU was further validated by dual-luciferase reporter assays. We found that mimic-320 significantly reduced the luciferase activity of the reporter vector containing wild-type (WT) OGRU, but not the reporter vector containing OGRU with mutated (MUT) miR-320-binding sites in 293T cells (Fig. 5i, j). miRNAs can bind their targets in an Ago2-dependent manner. Therefore, to determine whether OGRU was regulated by miR-320-3p in such a manner, we conducted anti-Ago2 RNA-binding protein immunoprecipitation (RIP) in MC3T3-E1 cells and found that miR-320-3p and OGRU were enriched in the anti-Ago2 group compared with the anti-IgG group, as detected by qRT-PCR (Fig. 5k). These data demonstrated that OGRU can act as a ceRNA for binding to miR-320-3p.

### miR-320-3p inhibits the osteoblast activity and matrix mineralization of MC3T3-E1 cells by targeting Hoxa10

**Fig. 6:**
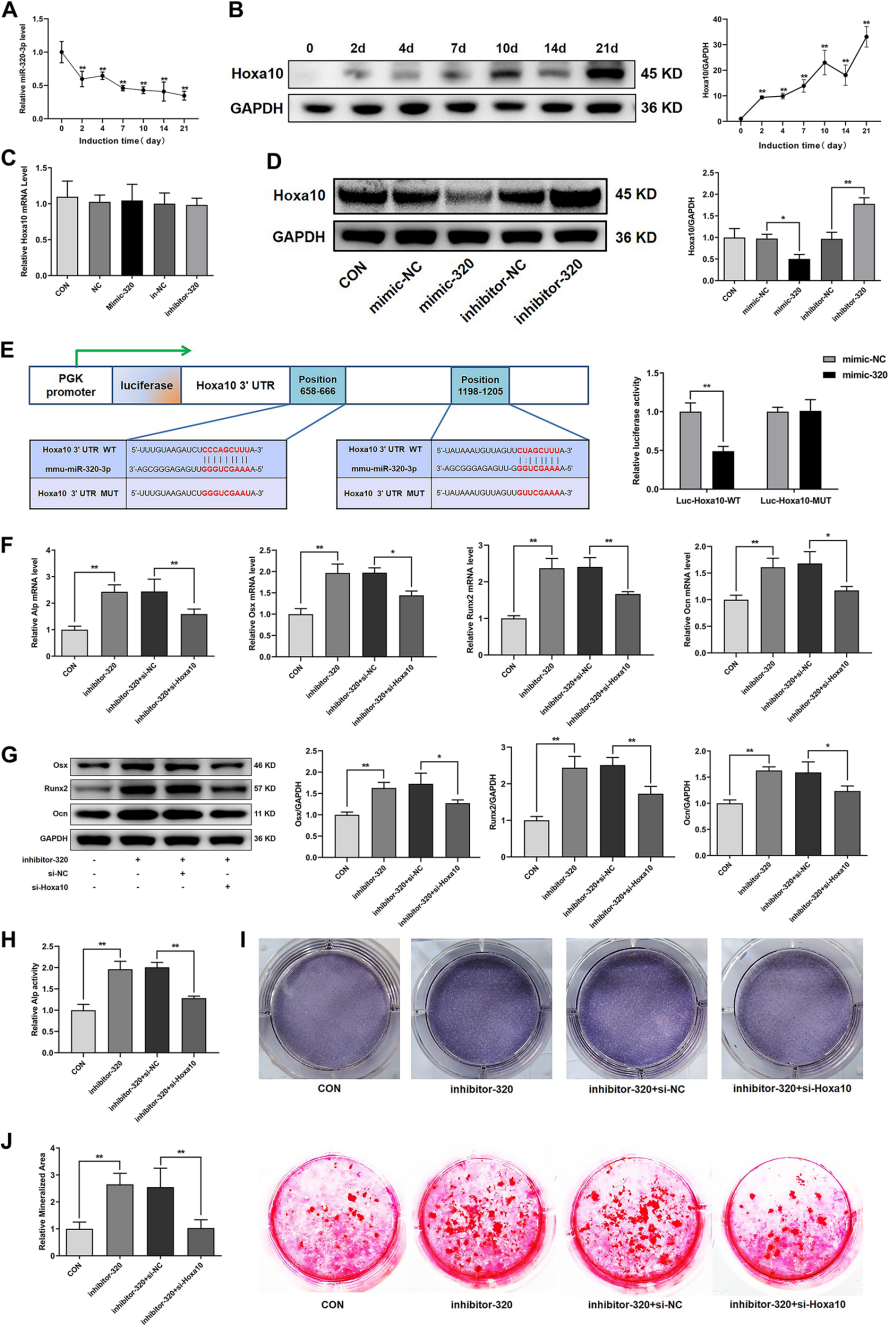
miR-320-3p inhibits the osteoblast activity and matrix mineralization of MC3T3-E1 cells by targeting Hoxa10. **a** qRT-PCR analysis of miR-320-3p levels during osteoblast differentiation of MC3T3-E1 cells (*n* = 3). **b** Western blot analysis of Hoxa10 protein expression during osteoblast differentiation of MC3T3-E1 cells (*n* = 3). **c** qRT-PCR analysis of Hoxa10 mRNA expression levels and **d** western blot analysis of Hoxa10 protein expression levels after treatment with mimic-320, inhibitor-320, or the corresponding controls (*n* = 3). **e** The relative luciferase activity of luciferase reporters containing the wild-type (WT) Hoxa10 3′UTR or the Hoxa10 3′UTR with a mutated (MUT) miR-320-binding site in 293T cells cotransfected with mimic-320 or its negative control was measured (*n* = 3). MC3T3-E1 cells were cotransfected with inhibitor-320 and si-Hoxa10 or its negative controls. qRT-PCR analysis of Alp, Osx, Runx2, and Ocn mRNA levels (**f**), western blot analysis of Osx, Runx2, and Ocn protein levels (**g**), relative Alp activity (**h**), representative images of Alp staining in MC3T3-E1 cells (**i**), and representative images of alizarin red staining in MC3T3-E1 cells are presented, and the relative areas of mineralization were quantified by Image J (**j**) (*n* = 3). All data are the mean ± SD. *P < 0.05, **P < 0.01.

A previous study confirmed that hsa-miR-320a-3p inhibits the osteoblast activity and matrix mineralization of human bone marrow-derived mesenchymal stem cells (hMSCs) by targeting Hoxa10, a critical regulator of osteogenesis^39,41,42^. Considering that mmu-miR-320-3p is the murine homolog of hsa-miR-320a-3p, and was predicted to target Hoxa10 by TargetScan, miRanda, and miRDB (Supplementary Table 3), we explored whether miR-320-3p could regulate the osteoblast activity and matrix mineralization of MC3T3-E1 cells in such a manner. During the osteoblast differentiation phase, miR-320-3p levels decreased gradually, whereas Hoxa10 protein levels increased (Fig. 6a, b). Moreover, miR-320-3p significantly influenced Hoxa10 protein expression, but had no effect on Hoxa10 mRNA levels, which indicated that miR-320-3p performs post-transcriptional regulation of Hoxa10 expression through mRNA translational repression but not mRNA cleavage (Fig. 6c, d). Next, we constructed luciferase reporters containing either the WT Hoxa10 3′UTR or the Hoxa10 3′UTR with mutated (MUT) miR-320-binding sites in 293T cells, and found that mimic-320 substantially inhibited the luciferase reporter activity of the WT Hoxa10 3′UTR, but not the MUT Hoxa10 3′UTR (Fig. 6e). Taken together, our results confirmed that Hoxa10 is the target of miR-320-3p.

To determine whether Hoxa10 is responsible for the regulatory effect of miR-320-3p on osteoblast activity and matrix mineralization, MC3T3-E1 cells were cotransfected with inhibitor-320 and si-Hoxa10 or its negative control, and were cultured in osteogenic medium. The results showed that si-Hoxa10 partially reduced the promotion of osteoblast activity and matrix mineralization induced by inhibitor-320, as determined by the mRNA or protein expression of osteogenic marker genes, Alp activity, Alp staining, and matrix mineralization (Fig. 6f–j).

### Hoxa10 is responsible for OGRU-mediated osteoblast differentiation and bone formation during unloading

**Fig. 7:**
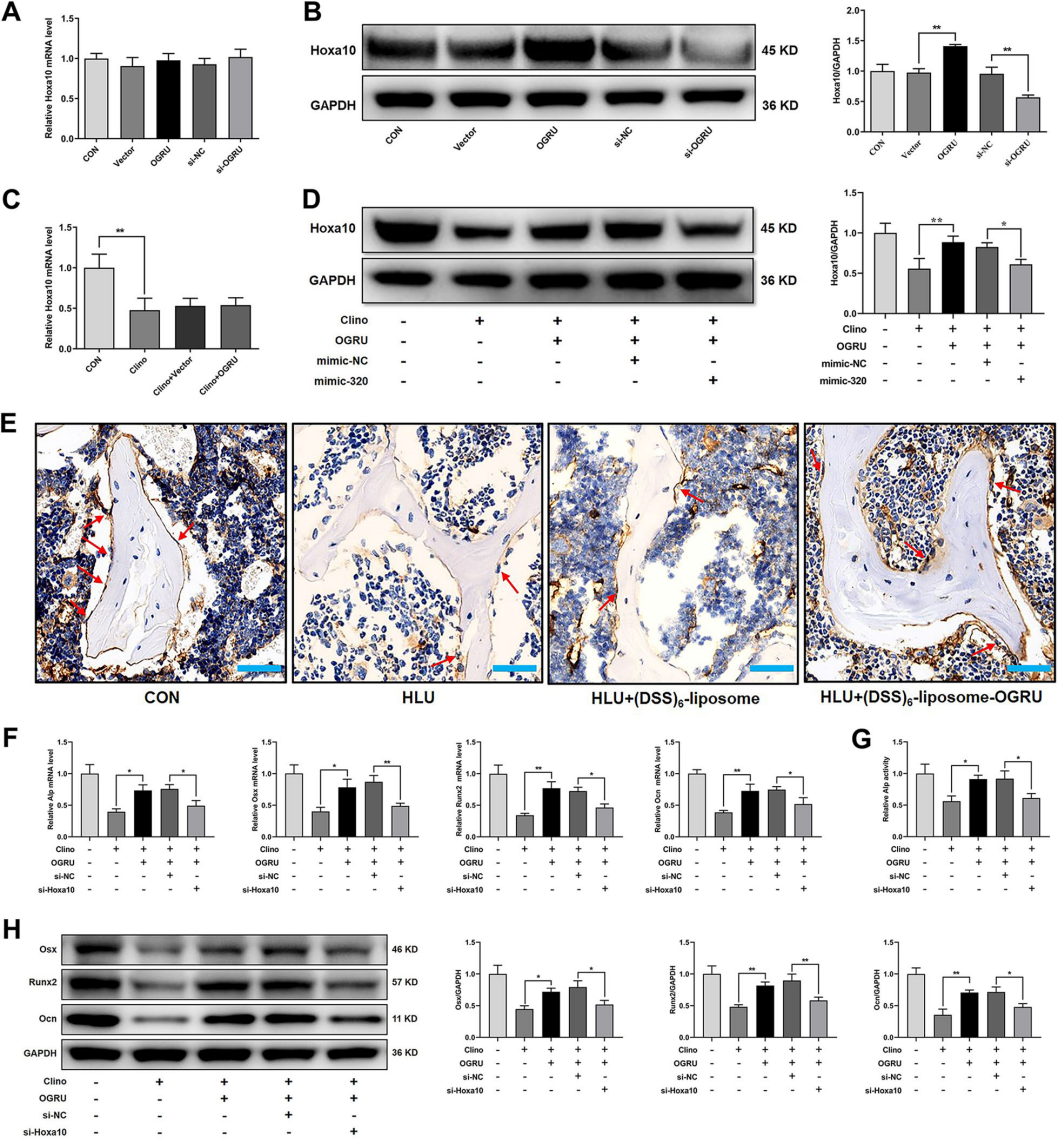
Hoxa10 is responsible for OGRU-mediated osteoblast differentiation and bone formation during unloading. **a** qRT-PCR analysis of Hoxa10 mRNA levels and **b** western blot analysis of Hoxa10 protein levels after treatment with pcDNA3.1(+)–OGRU, si-OGRU, or the corresponding controls in MC3T3-E1 cells (*n* = 3). **c** Hoxa10 mRNA levels were measured by qRT-PCR, and **d** Hoxa10 protein expression was measured by western blotting after MC3T3-E1 cells were transfected with pcDNA3.1(+)–OGRU alone or pcDNA3.1(+)–OGRU plus mimic-320, and subjected to clinorotation unloading for 48 h (*n* = 3). **e** Representative images of Hoxa10 immunohistochemical staining in the femurs of mice from the indicated groups (*n* = 5). **f** qRT-PCR analysis of Alp, Osx, Runx2, and Ocn mRNA levels, **g** western blot analysis of Osx, Runx2, and Ocn protein expression, and **h** the relative Alp activity after MC3T3-E1 cells were cotransfected with pcDNA3.1(+)–OGRU and si-Hoxa10 or its negative controls, and subjected to clinorotation unloading for 48 h (*n* = 3). All data are the mean ± SD. *P < 0.05, **P < 0.01.

Since OGRU interacted with miR-320-3p, and Hoxa10 was the target of miR-320-3p, we examined whether OGRU could positively regulate the expression of Hoxa10. Our results demonstrated that OGRU promoted the protein expression of Hoxa10, but did not influence the mRNA expression of Hoxa10 under normal conditions (Fig. 7a, b). Besides, OGRU partially reversed the reduction in Hoxa10 protein expression induced by clinorotation unloading in MC3T3-E1 cells through miR-320-3p, but had no effect on the mRNA expression of Hoxa10 under clinorotation-unloading conditions (Fig. 7c, d). Immunohistochemical staining of Hoxa10 also indicated that OGRU partially reversed the reduction in Hoxa10 protein expression induced by HLU (Fig. 7e). In addition, the effect of the enhanced osteoblast differentiation induced by OGRU during unloading was reversed by si-Hoxa10, as evidenced by the mRNA levels of Alp, Osx, Runx2, and Ocn, the protein expression of Osx, Runx2, and Ocn, and Alp activity (Fig. 7f–h).

## Discussion

Skeletal unloading by long-duration space flight and extended bed rest can induce disuse osteopenia, which is regulated by various cytokines, hormones, and miRNAs^14,43^. However, scant information addresses the role of lncRNAs in unloading-induced bone loss. In this study, we identified a novel unloading-sensitive lncRNA, OGRU, and confirmed its direct impact on osteoblast differentiation during unloading in vitro. Moreover, bone-targeted OGRU counteracted bone loss in HLU mice. The mechanistic studies showed that OGRU sponges miR-320-3p to promote osteoblast differentiation and bone formation by upregulating the protein expression of Hoxa10. Thus, our research was the first to elucidate the role of the OGRU/miR-320-3p/Hoxa10 pathway in the pathophysiological process of unloading-induced bone loss.

Multiple lncRNAs have been identified as important regulators of osteoblast differentiation and bone formation via a variety of mechanisms dependent on their subcellular location^44^. For instance, the lncRNA Bmncr can alleviate bone loss and bone marrow fat accumulation during aging by facilitating the assembly of the TAZ and RUNX2/PPARG transcriptional complex^29^. Moreover, the lncRNA MEG3 can activate BMP4 transcription by dissociating SOX2 from the BMP4 promoter, and thereby promote osteoblast differentiation of MSCs^28^. An additional study reported that the lncRNA PGC1β-OT1 can stimulate osteoblast differentiation of progenitor cells in vitro and in vivo by antagonizing the function of miR-148a-3p^45^. In particular, the lncRNA H19 (H19) is a key regulator of osteoblast function, and is involved in the development of disuse osteoporosis, suggesting that lncRNAs may play a key role in unloading-induced bone loss^31,32,46,47^. Our previous work found many lncRNAs that are significantly differentially expressed in MC3T3-E1 cells under clinorotation-unloading condition for 48 h^35^. Further exploration identified a novel unloading-sensitive lncRNA, OGRU, which promotes the osteoblast activity and matrix mineralization, and attenuates the osteogenic impairment of MC3T3-E1 cells induced by clinorotation unloading. To further determine whether OGRU can rescue unloading-induced bone loss in vivo, pcDNA3.1(+)–OGRU was delivered via (DSS)_6_–liposomes to bone-formation surfaces in HLU mice. In this regard, OGRU can promote bone formation, and can be used as a therapeutic target against unloading-induced bone loss. However, it has been reported that unloading-induced bone loss is caused by both decreased bone formation and increased bone resorption. In this study, OGRU was delivered only to bone-formation surfaces in HLU mice; thus, its effects on bone resorption have not been elucidated.

To date, many RNA transcripts, such as lncRNAs and circular RNAs, have been thoroughly characterized as ceRNAs for microRNA binding^48,49^. Here, we found that OGRU contains binding sites, and can interact with miR-320-3p in the Ago2–RISC complex. Previously, it has been reported that miR-320 is a critical regulator of tumorigenesis, inflammation, and substance metabolism^50–53^. In addition, hsa-miR-320a-3p inhibits osteoblast differentiation of hMSCs^39^. However, scant information addresses the role of mmu-miR-320-3p in osteoblast differentiation and bone formation, especially during unloading. In this study, in vitro and in vivo experiments strongly suggested that miR-320-3p is unloading-sensitive, and that its levels can be decreased by OGRU during unloading. Furthermore, we demonstrated that miR-320-3p inhibits the osteoblast activity and matrix mineralization of MC3T3-E1 cells by targeting Hoxa10.

Hoxa10, a member of the homeobox gene family, can participate in the development and growth of multiple systems, as indicated by its critical roles in male and female fertility, liver tumorigenesis, gastric cancer invasion, and leukemogenesis^54–57^. Moreover, multiple lines of evidence have shown that Hoxa10 is necessary for the global patterning of the mammalian skeleton^58–60^. Furthermore, Hoxa10 can promote osteoblast differentiation through the activation of Runx2 and directly regulate osteoblast differentiation genes, including the alkaline phosphatase, osteocalcin, and bone sialoprotein genes, and this process can be regulated by the Pbx1 complex^41,42,61,62^. In ovariectomized mice, Hoxa10 protein levels were significantly reduced by miR-705, but Hoxa10 mRNA levels were not significantly changed^63^. Here, we reported that both the protein and mRNA levels of Hoxa10 were decreased by unloading in vitro and in vivo, but only the decrease in Hoxa10 protein levels was ameliorated by the OGRU/miR-320-3p axis. Accordingly, we speculated that Hoxa10 can also be regulated by other mechanisms at the transcriptional and epigenetic levels during unloading.

**Fig. 8:**
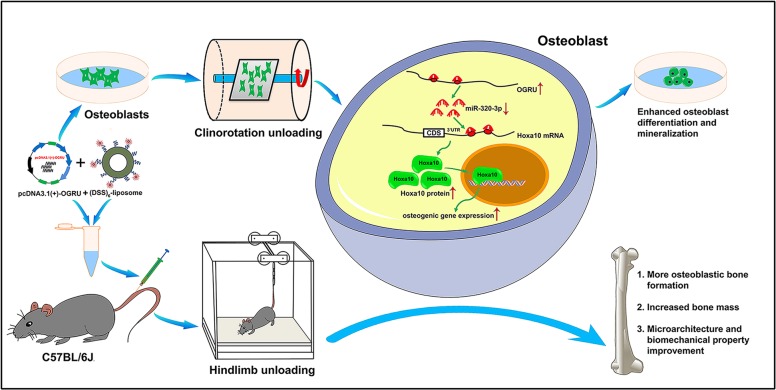
A schematic illustrating the molecular mechanisms of OGRU-regulated unloading-induced bone loss. OGRU significantly promotes Hoxa10 protein expression by functioning as a miR-320-3p sponge, which results in enhanced osteoblast differentiation and mineralization in vitro, and more osteoblastic bone formation, increased bone mass and trabecular microarchitecture, and biomechanical property improvements in vivo during mechanical unloading condition.

In summary, our findings uncover a novel unloading-sensitive lncRNA OGRU, which is a critical regulator of osteoblast activity and matrix mineralization, and attenuates osteogenic decline by functioning as a miR-320-3p sponge to stimulate Hoxa10 protein expression. Correspondingly, bone-targeted OGRU can effectively counteract unloading-induced bone loss, indicating that OGRU may be a promising therapeutic target for unloading-induced bone loss and disuse osteoporosis (Fig. 8).

## Materials and methods

### Cell culture and in vitro differentiation

The murine preosteoblast cell line MC3T3-E1 clone 14 was purchased from the Cell Bank of the Chinese Academy of Sciences (Shanghai, China) and cultured in alpha-modified Eagle’s medium (α-MEM, Gibco, Grand Island, NY, USA) supplemented with 10% FBS (HyClone Laboratories, Logan, UT, USA) and 1% penicillin and streptomycin (Thermo Fisher Scientific, Waltham, MA, USA) in an atmosphere of 5% CO_2_ and 95% humidity at 37 °C. The culture medium was changed every 2 days. Cells beyond passage 15 were not used for the experiments. For osteoblast differentiation, MC3T3-E1 cells at an appropriate confluency were induced by the addition of 100 nM dexamethasone, 10 mM β-glycerophosphate, and 50 μg/ml ascorbic acid to the culture medium. Cell experiments were repeated three times (*n* = 3), and the investigator knew the group allocation during the experiment.

### 2D clinorotation

A 2D clinostat (developed by the China Astronaut Research and Training Center, Beijing, China) was selected to simulate the unloading environment in vitro, and related experiments were performed according to the procedure described previously^12,64^. Briefly, MC3T3-E1 cells were seeded in 25-cm^2^ culture flasks at a density of 5 × 10^5^ cells per flask. After cell adherence, the culture flasks were filled with osteogenic medium, ensuring that air bubbles were removed. Then, the culture flasks were fixed to the 2D clinostat, and rotated around the horizontal axis at 24 rpm for 24, 48, and 72 h. The CON groups were placed in a similar incubator without clinorotation.

### HLU mice and experimental design

Male C57BL/6J mice purchased from the Animal Center of Air Force Medical University (Xi’an, China) were housed under standard conditions (22 °C, 50–55% humidity, and a 12-h light/12-h dark cycle). For assessment of OGRU overexpression efficiency, targeting ability of (DSS)_6_–liposome delivery system to bone-formation surfaces, and toxicity in vivo, ten 6-month-old male C57BL/6J mice were randomly divided into two groups by random scale, as follows: (1) PBS and (2) (DSS)_6_–liposome–OGRU–GFP (*n* = 5). The organs of mice (hearts, livers, lungs, kidneys, and bilateral femurs and tibias) were collected for analysis 3 days after a single injection of (DSS)_6_–liposome–OGRU–GFP or PBS.

For assessment of the protective effect of bone-targeted OGRU on unloading-induced bone loss in HLU mice, HLU was performed to obtain a mouse model with unloading-induced bone loss, as previously reported^12,65^. Briefly, 6-month-old mice were suspended at a 30° angle with the floor by the tail, which allowed the mice to move and access food and water freely. As illustrated in Fig. 3b, twenty 6-month-old male C57BL/6J mice were randomly divided into four groups by random scale, as follows: (1) CON, (2) HLU, (3) HLU + (DSS)_6_–liposome, and (4) HLU + (DSS)_6_–liposome–OGRU (*n* = 5). Before HLU, either pcDNA3.1(+)–OGRU (2 mg/kg body weight) carried by (DSS)_6_–liposomes or (DSS)_6_–liposomes alone were administered by three consecutive intravenous injections. The bilateral femurs and tibias of the mice were collected for bone analysis. Specifically, the left tibias of mice were used for RNA extraction, while the right tibias were used for double-calcein-labeling assay, and the right femurs were used for histological analysis after micro-CT analysis, while the left femurs were used for biomechanical testing. In animal experiments, the researchers were blinded to the group allocation during the experiments and when assessing the outcome. All experimental protocols were approved by the Animal Care Committee of Air Force Medical University.

### RNA extraction and quantitative real-time polymerase chain reaction (qRT-PCR) analysis

The total RNA of cells or left tibias was isolated using RNAiso Plus according to the manufacturer’s protocol (TaKaRa, Dalian, China). The total RNA was reverse-transcribed to complementary DNA (cDNA) using a Prime Script™ RT Master Mix Kit (TaKaRa, Dalian, China) and the following procedure: 37 °C for 15 min, 85 °C for 5 s, and holding at 4 °C. For miRNA expression analysis, miRNA was reverse-transcribed using a Mir-X miRNA First-Strand Synthesis Kit (Clontech, Palo Alto, CA, USA) and the following procedure: incubation at 37 °C for 1 h, termination at 85 °C for 5 min, and holding at 4 °C. qRT-PCR reactions were performed using a SYBR^®^ Premix Ex Taq™ II Kit (TaKaRa, Dalian, China) and a CFX96 real-time PCR detection system (Bio-Rad Laboratories, Hercules, CA, USA). The expression of lncRNAs and mRNAs was analyzed using the following cycling program: denaturation for 2 min at 95 °C, 40 cycles of annealing for 10 s at 95 °C, and elongation for 40 s at 60 °C. GAPDH was used as the reference gene. The expression of miRNA was analyzed using the following cycling program: 30 s at 95 °C, 40 cycles of 5 s at 95 °C, and 30 s at 60 °C. U6 was used as the reference gene. The primer sequences used are shown in Supplementary Table 4.

### Protein isolation and western blotting analysis

Total protein was extracted from cells using M-PER Mammalian Protein Extraction Reagent (Thermo Fisher Scientific, Waltham, MA, USA) supplemented with protease inhibitor cocktail (Roche, Mannheim, Germany), and was quantitated by a Pierce™ BCA Protein Assay Kit (Thermo Fisher Scientific, Waltham, MA, USA) following the manufacturer’s protocol. After protein samples were heated at 70 °C for 10 min, appropriate amounts of samples containing protein along with LDS sample buffer, reducing agent (Thermo Fisher Scientific, Waltham, MA, USA), and double-distilled H_2_O were subjected to electrophoresis on NuPAGE™ Bis-Tris Protein Gels (Thermo Fisher Scientific, Waltham, MA, USA). After electrophoresis, the separated proteins were transferred to polyvinylidene difluoride (PVDF) membranes with a constant current of 250 mA for 2 h at 4 °C. Then, PVDF membranes were blocked with 5% nonfat milk in TBST for 2 h at room temperature, and incubated with primary antibodies specific for Osx (1:1000, Abcam ab93876, USA), Runx2 (1:1000, Cell Signaling Technology #12556S, USA), Ocn (1:2000, Abcam ab93876, USA), GAPDH (1:5000, Proteintech 60004-1-Ig, USA), and Hoxa10 (1:1000, Santa Cruz Biotechnology, sc-271428, USA) in primary antibody dilution buffer (Beyotime, Shanghai, China) overnight at 4 °C. Next, PVDF membranes were incubated with peroxidase-conjugated goat anti-rabbit IgG or goat anti-mouse IgG (1:5000, ZSGB-BIO, Beijing, China) for 1 h at room temperature, and were visualized by chemiluminescence reagent (Millipore, Billerica, MA, USA). GAPDH served as the reference gene. The relative quantity of protein expression was analyzed with the Image J software.

### 5′ and 3′ rapid amplification of cDNA ends (RACE)

The transcription initiation and termination sites of OGRU were detected by 5′ and 3′ RACE using a SMARTer^®^ RACE 5′/3′ Kit (Clontech, Palo Alto, CA, USA) according to the manufacturer’s instructions. Briefly, RNA was extracted from MC3T3-E1 cells, and 3′- and 5′-RACE-Ready cDNA was synthesized using SMARTScribe Reverse Transcriptase, as follows: 42 °C for 90 min and 70 °C for 10 min. Amplification was performed as follows: five cycles at 94 °C for 30 s and 72 °C for 3 min, five cycles at 94 °C for 30 s, 70 °C for 30 s, and 72 °C for 3 min, and twenty cycles at 94 °C for 30 s, 68 °C for 30 s, and 72 °C for 3 min. The obtained gel products were extracted and cloned into the pEASY cloning vector for sequencing. The gene-specific primers used are listed in Supplementary Table 5.

### Northern blot analysis

The total RNA extracted from cells combined with 5× formaldehyde gel electrophoresis buffer, 37% formaldehyde, and formamide was heated at 65 °C for 15 min and cooled on ice for 5 min. RNA-loading buffer was then added. Samples were subjected to formaldehyde gel electrophoresis at 50 V for 2 h, and transferred to a nylon membrane. After prehybridization for 3 h at 42 °C, the membrane was hybridized with a DIG-labeled probe complementary to OGRU at 42 °C for 16 h. The membrane was visualized using a Tanon 4600 (Tanon, Shanghai, China). The DIG-labeled probe sequence complementary to OGRU was as follows: TTTGGTTGACTTCCCTGATACTTCAGAAAGATAAGAAAATGAACTCTACTCTCTTGCTTCTGGATCTTTTGTTCCCCTCTGTCTCCCCATTCCTTTCCTCCAACTCTCCACATGTTAATGGCTGGCCTCTCCTTATCTACTCTTTCTCTCTGCCTTTCTCGACTCTAGGACCCTCTTAACTC.

### Cell transfection

Cells were transfected with the miR-320-3p mimic (40 nM) and miR-320-3p inhibitor (80 nM) or their corresponding negative controls (GenePharma, Shanghai, China) using a Lipofectamine 2000 kit (Thermo Fisher Scientific, Waltham, MA, USA) following the manufacturer’s instructions. siRNAs (80 nM) specific for lnc-OGRU or Hoxa10 (GenePharma, Shanghai, China) and pcDNA3.1(+)–OGRU (200 ng/μl) (GeneCreate, Wuhan, China) were transfected into cells using a Lipofectamine 3000 kit (Thermo Fisher Scientific, Waltham, MA, USA). The sequences of the siRNAs are shown in Supplementary Table 6.

### Alkaline phosphatase activity assay

Total protein extracted from cells was utilized to measure Alp activity using an alkaline phosphatase assay kit (Nanjing Jiancheng Technological, China) following the manufacturer’s protocol. The protein concentration was measured by the Pierce™ BCA Protein Assay Kit (Thermo Fisher Scientific, Waltham, MA, USA). Phenol (0.02 mg/ml) was used as the standard solution, and double-distilled H_2_O was used as the blank control solution. Alp activity was defined as the amount of phenol produced after 1 g of protein reacted with the substrate for 15 min at 37 °C.

### Alkaline phosphatase staining

After MC3T3-E1 cells were cultured in osteogenic medium for 7 days, alkaline phosphatase staining was performed. Briefly, cells in six-well plates were fixed with 4% paraformaldehyde for 15 min, and stained with a BCIP/NBT Alp Color Development kit (Beyotime, Shanghai, China) for 30 min at room temperature according to the manufacturer’s instructions. The whole procedure was performed in the dark. Images were acquired by a digital camera.

### Alizarin red staining

MC3T3-E1 cells cultured in osteogenic medium for 21 days were washed with DPBS three times and fixed with ice-cold 70% ethanol for 40 min at 4 °C. After three washes with double-distilled H_2_O, cells were stained in 1% alizarin red S (Sigma, St. Louis, MO, USA) for 15 min at room temperature. Next, cells were rinsed five times with double-distilled H_2_O, and the stained cells were imaged by a digital camera.

### Micro-CT analysis

Dissected right femurs were fixed with 4% paraformaldehyde for 2 days and scanned with a micro-CT imaging system (Siemens, Germany) with a spatial resolution of 10.44 μm (80 kV, 500 mA, 800-ms integration time), as previously reported^66^. The region of interest (ROI) selected was a 2.5 × 2.5 × 3-mm^3^ cube ~1.5 mm from the growth plate in the distal femurs. A 3D reconstruction of the ROI was used to determine the bone mineral density (BMD), relative bone volume (BV/TV), trabecular bone thickness (Tb.Th), trabecular bone number (Tb.N), the ratio of bone surface to bone volume (BS/BV), trabecular bone separation (Tb.Sp), trabecular bone pattern factor (Tb.PF), and cortical thickness (Ct.Th) values with COBRA software. The threshold used for distinguishing mineralized and nonmineralized tissue in the micro-CT evaluations was 800 HU.

### Histological analysis

After subjecting to micro-CT analysis, right femurs were decalcified in 10% EDTA for 3 weeks, and embedded in paraffin before sectioning (5 μm, longitudinally oriented). Bone sections were subjected to H&E and Goldner’s trichrome staining according to the standard protocol. The paraffin sections of the heart, liver, lung, and kidney were subjected to H&E staining according to the standard protocol. For immunohistochemical staining, the primary antibodies used were specific for Ocn (1:100, Abcam ab93876, USA) or Hoxa10 (1:100, Santa Cruz Biotechnology sc-271428, USA). Sections were examined with a microscope (Eclipse E100, Nikon, Japan). For immunofluorescence staining of GFP or Ocn, bone sections were stained with individual primary antibodies to GFP (1:1000, Abcam ab1218, USA) or Ocn (1:200, Abcam ab93876, USA). Then, bone sections were stained with FITC-conjugated anti-mouse IgG (1:100, Proteintech SA00003-1, USA) or CY3-conjugated anti-rabbit IgG (1:100, Proteintech SA00009-2, USA). Nuclei were stained with DAPI. Images were acquired under a fluorescence microscope (Eclipse E100, Nikon, Japan).

### Double-calcein-labeling assay

Mice received intraperitoneal injection of calcein (8 mg/kg body weight, Sigma, St. Louis, MO, USA) 10 days and 3 days before sacrifice. After the collected right tibias were fixed with 4% paraformaldehyde for 2 days and embedded in polymethylacrylate, every sample was serially cut into three 50-μm-thick sections with a hard tissue-slicing machine (SP1600, Leica, Germany) in the dark, and double-calcein labeling was imaged by confocal microscopy (LSM800, ZEISS, Germany). The distance between two fluorescence-labeled lines as measured three times with Image J software was used to evaluate the mineral apposition rate (MAR) of trabecular bone.

### Biomechanical testing

The dissected left femurs were wrapped with gauze dipped in saline and stored at −80 °C. A three-point bending test was performed using an electromechanical material testing machine (Bose, USA). The femurs were placed on a supporter with two points with a 8-mm span distance, and a load was applied vertically to the midshaft of the femurs at a constant displacement rate of 0.02 mm/s until fracture. Then, the lengths of the external major and minor axes, and the thickness of the cortical bone at the fracture site, were measured using a Vernier caliper. The values of the structural properties were calculated according to the load-deflection curve, and included the maximum load, stiffness, and elastic modulus^67^.

### RNA fluorescence in situ hybridization

Cells were fixed with 4% paraformaldehyde for 20 min at room temperature. Then, hybridization solution containing a FAM-labeled OGRU probe (5′-TGAAGTATCAGGGAAGTCAACCAA-3′) was added, and cells were hybridized overnight at 37 °C. DAPI was used to stain nuclei. Images were acquired under a fluorescence microscope (Eclipse E100, Nikon, Japan).

### Nuclear–cytoplasmic fractionation

Cytoplasmic and nuclear RNA were isolated from MC3T3-E1 cells with a PARIS™ Kit (Thermo Fisher Scientific, Waltham, MA, USA) following the manufacturer’s instructions. Briefly, 400 µl of ice-cold Cell Disruption Buffer was added to resuspend 10^6^ MC3T3-E1 cells, and then the samples were centrifuged for 5 min at 4 °C and 500 × *g* to separate the nuclear and cytoplasmic cell fractions. In addition, the supernatant (cytoplasmic fraction) was put in a fresh RNase-free tube at 4 °C, while the nuclear pellet was lysed by 400 µl of ice-cold Cell Disruption Buffer. After that, the nuclear and cytoplasmic cell fractions will be used for RNA isolation. Finally, RNA samples were reverse-transcribed to cDNA, and qRT-PCR was performed to detect OGRU expression in the nucleus and cytoplasm, as described above. 45S rRNA (primarily in the nucleus) and 12S rRNA (primarily in the cytoplasm) were used as controls.

### Plasmid constructs and luciferase activity assays

The luciferase constructs containing OGRU-WT and Hoxa10 3′UTR WT, or OGRU-MUT and Hoxa10 3′UTR MUT, were generated by inserting a PCR fragment containing the predicted or mutated binding sites of miR-320-3p into the pmirGLO vector between the SacI and XhoI sites. The luciferase construct and the miR-320-3p mimic or its negative control were cotransfected into 293T cells. Luciferase activity was measured 24 h post transfection using the dual-luciferase reporter assay system (Promega, Madison, WI, USA). Firefly luciferase activity was normalized to renilla luciferase activity.

### RNA-binding protein immunoprecipitation (RIP)

RIP experiments were performed using a Magna RIP™ RNA-Binding Protein Immunoprecipitation Kit (Millipore, Billerica, MA, USA) according to the manufacturer’s instructions. The antibodies used were anti-Ago2 (Abcam ab32381, USA) and anti-IgG (Millipore PP64B, USA). qRT-PCR was used to detect OGRU and miR-320-3p expression among the precipitated RNAs.

### Statistical analysis

All data are expressed as the means ± SDs, and were analyzed using SPSS Statistics 22.0. Two-group comparisons were performed using Student’s *t* test, and multiple group comparisons were analyzed by one-way ANOVA followed by the LSD post hoc test. *P* < 0.05 was considered significant.

## Supplementary information

Supplementary Figure 1

Supplementary Figure 2

Supplementary Figure 3

Supplementary Figure 4

Supplementary Figure 5

Supplementary Figure 6

Supplementary Figure 7

Supplementary Figure 8

Supplemental Figure Legends and Tables

## References

[1] Lian JB (2012). MicroRNA control of bone formation and homeostasis. Nat. Rev. Endocrinol..

[2] Rodan GA, Martin TJ (2000). Therapeutic approaches to bone diseases. Science.

[3] Ahn AC, Grodzinsky AJ (2009). Relevance of collagen piezoelectricity to “Wolff’s Law”: a critical review. Med. Eng. Phys..

[4] Vico L (2000). Effects of long-term microgravity exposure on cancellous and cortical weight-bearing bones of cosmonauts. Lancet.

[5] Cabahug-Zuckerman P (2016). Osteocyte apoptosis caused by hindlimb unloading is required to trigger osteocyte RANKL production and subsequent resorption of cortical and trabecular bone in mice femurs. J. Bone Min. Res..

[6] Wang X (2013). miR-214 targets ATF4 to inhibit bone formation. Nat. Med..

[7] Xu Z (2017). The regulation of iron metabolism by hepcidin contributes to unloading-induced bone loss. Bone.

[8] Sakata T (2004). Skeletal unloading induces resistance to insulin-like growth factor-I (IGF-I) by inhibiting activation of the IGF-I signaling pathways. J. Bone Min. Res..

[9] Morey-Holton ER, Globus RK (1998). Hindlimb unloading of growing rats: a model for predicting skeletal changes during space flight. Bone.

[10] Rutkovskiy A, Stensløkken KO, Vaage IJ (2016). Osteoblast differentiation at a glance. Med. Sci. Monit. Basic Res..

[11] Herranz R, Valbuena MA, Manzano A, Kamal KY, Medina FJ (2015). Use of microgravity simulators for plant biological studies. Methods Mol. Biol..

[12] Wang Y (2018). MicroRNA-139-3p regulates osteoblast differentiation and apoptosis by targeting ELK1 and interacting with long noncoding RNA ODSM. Cell Death Dis..

[13] Arfat Y (2018). miR-208a-3p suppresses osteoblast differentiation and inhibits bone formation by targeting ACVR1. Mol. Ther. Nucleic Acids.

[14] Malaval L (2008). Bone sialoprotein plays a functional role in bone formation and osteoclastogenesis. J. Exp. Med..

[15] Hattori M (2004). Finishing the euchromatic sequence of the human genome. Nature.

[16] Wang Y (2015). The long noncoding RNA lncTCF7 promotes self-renewal of human liver cancer stem cells through activation of Wnt signaling. Cell Stem Cell.

[17] Mercer TR, Dinger ME, Mattick JS (2009). Long non-coding RNAs: insights into functions. Nat. Rev. Genet..

[18] Rinn JL (2007). Functional demarcation of active and silent chromatin domains in human HOX loci by noncoding RNAs. Cell.

[19] Morris KV, Santoso S, Turner AM, Pastori C, Hawkins PG (2008). Bidirectional transcription directs both transcriptional gene activation and suppression in human cells. PLoS Genet..

[20] Dinger ME (2008). Long noncoding RNAs in mouse embryonic stem cell pluripotency and differentiation. Genome Res..

[21] Nagano T (2008). The Air noncoding RNA epigenetically silences transcription by targeting G9a to chromatin. Science.

[22] Pandey RR (2008). Kcnq1ot1 antisense noncoding RNA mediates lineage-specific transcriptional silencing through chromatin-level regulation. Mol. Cell..

[23] Wang X (2008). Induced ncRNAs allosterically modify RNA-binding proteins in cis to inhibit transcription. Nature.

[24] Liang H (2018). LncRNA PFL contributes to cardiac fibrosis by acting as a competing endogenous RNA of let-7d. Theranostics.

[25] Chen DL (2017). Long non-coding RNA UICLM promotes colorectal cancer liver metastasis by acting as a ceRNA for microRNA-215 to regulate ZEB2 expression. Theranostics.

[26] Ji-hang Yuan, F. Y. et al. A long noncoding RNA activated by TGF-b promotes the invasion-metastasis cascade in hepatocellular carcinoma. *Cancer Cell.* **25**, 666–681 (2014). 10.1016/j.ccr.2014.03.010 24768205

[27] Hu WL (2018). GUARDIN is a p53-responsive long non-coding RNA that is essential for genomic stability. Nat. Cell Biol..

[28] Zhuang W (2015). Upregulation of lncRNA MEG3 promotes osteogenic differentiation of mesenchymal stem cells from multiple myeloma patients by targeting BMP4 transcription. Stem Cells.

[29] Li CJ (2018). Long noncoding RNA Bmncr regulates mesenchymal stem cell fate during skeletal aging. J. Clin. Invest..

[30] Jia B (2019). Long noncoding RNA LINC00707 sponges miR-370-3p to promote osteogenesis of human bone marrow-derived mesenchymal stem cells through upregulating WNT2B. Stem Cell Res. Ther..

[31] Li B (2017). LncRNA-H19 modulates Wnt/β-catenin signaling by targeting Dkk4 in hindlimb unloaded rat. Orthop. Surg..

[32] Li B (2018). Overexpression of DNMT1 leads to hypermethylation of H19 promoter and inhibition of Erk signaling pathway in disuse osteoporosis. Bone.

[33] Zhang G (2012). A delivery system targeting bone formation surfaces to facilitate RNAi-based anabolic therapy. Nat. Med..

[34] Yang K, Miron RJ, Bian Z, Zhang YF (2018). A bone-targeting drug-delivery system based on Semaphorin 3A gene therapy ameliorates bone loss in osteoporotic ovariectomized mice. Bone.

[35] Hu Z (2017). Genome‑wide analysis and prediction of functional long noncoding RNAs in osteoblast differentiation under simulated microgravity. Mol. Med. Rep..

[36] Zhang Y (2017). MicroRNA-221 is involved in the regulation of osteoporosis through regulates RUNX2 protein expression and osteoblast differentiation. Am. J. Transl. Res..

[37] Hao C (2016). MiR-708 promotes steroid-induced osteonecrosis of femoral head, suppresses osteogenic differentiation by targeting SMAD3. Sci. Rep..

[38] Zhang GP (2017). MicroRNA-98 regulates osteogenic differentiation of human bone mesenchymal stromal cells by targeting BMP2. J. Cell Mol. Med..

[39] Huang J (2016). MicroRNA-320a regulates the osteogenic differentiation of human bone marrow-derived mesenchymal stem cells by targeting HOXA10. Cell Physiol. Biochem..

[40] Yeh CH, Jin L, Shen F, Balian G, Li XJ (2016). miR-221 attenuates the osteogenic differentiation of human annulus fibrosus cells. Spine J..

[41] Gordon JA (2010). Pbx1 represses osteoblastogenesis by blocking Hoxa10-mediated recruitment of chromatin remodeling factors. Mol. Cell Biol..

[42] Hassan MQ (2007). HOXA10 controls osteoblastogenesis by directly activating bone regulatory and phenotypic genes. Mol. Cell Biol..

[43] Rucci N (2015). Lipocalin 2: a new mechanoresponding gene regulating bone homeostasis. J. Bone Min. Res..

[44] Zhang J, Hao X, Yin M, Xu T, Guo F (2019). Long non-coding RNA in osteogenesis: a new world to be explored. Bone Jt. Res.

[45] Yuan H, et al. A novel long noncoding RNA PGC1β-OT1 regulates adipocyte and osteoblast differentiation through antagonizing miR-148a-3p. *Cell Death Differ.* **26**, 2029–2045 (2019). 10.1038/s41418-019-0296-7 PMC6748127 30728459

[46] Huang Y, Zheng Y, Jia L, Li W (2015). Long noncoding RNA H19 promotes osteoblast differentiation via TGF-β1/Smad3/HDAC signaling pathway by deriving miR-675. Stem Cells.

[47] Liang WC (2016). H19 activates Wnt signaling and promotes osteoblast differentiation by functioning as a competing endogenous RNA. Sci. Rep..

[48] Hansen TB (2013). Natural RNA circles function as efficient microRNA sponges. Nature.

[49] Salmena L, Poliseno L, Tay Y, Kats L, Pandolfi PP (2011). A ceRNA hypothesis: the Rosetta Stone of a hidden RNA language. Cell.

[50] Lopetuso LR (2018). IL-33 promotes recovery from acute colitis by inducing miR-320 to stimulate epithelial restitution and repair. Proc. Natl Acad. Sci. USA.

[51] Zhang LL (2018). Interference with lactate metabolism by mmu-miR-320-3p via negatively regulating GLUT3 signaling in mouse Sertoli cells. Cell Death Dis..

[52] Zhu H (2018). Neuropilin-1 regulated by miR-320 contributes to the growth and metastasis of cholangiocarcinoma cells. Liver Int..

[53] Wu YY (2014). miR-320 regulates tumor angiogenesis driven by vascular endothelial cells in oral cancer by silencing neuropilin 1. Angiogenesis.

[54] Satokata I, Benson G, Maas R (1995). Sexually dimorphic sterility phenotypes in Hoxa10-deficient mice. Nature.

[55] Shao M (2018). LncHOXA10 drives liver TICs self-renewal and tumorigenesis via HOXA10 transcription activation. Mol. Cancer.

[56] Shao L (2018). Methylation of the HOXA10 promoter directs miR-196b-5p-dependent cell proliferation and invasion of gastric cancer cells. Mol. Cancer Res..

[57] Johansen S, Brenner AK, Bartaula-Brevik S, Reikvam H, Bruserud Ø (2018). The possible importance of β3 integrins for leukemogenesis and chemoresistance in acute myeloid leukemia. Int J. Mol. Sci..

[58] Favier B (1996). Functional cooperation between the non-paralogous genes Hoxa-10 and Hoxd-11 in the developing forelimb and axial skeleton. Development.

[59] Wellik DM, Capecchi MR (2003). Hox10 and Hox11 genes are required to globally pattern the mammalian skeleton. Science.

[60] Vinagre T (2010). Evidence for a myotomal Hox/Myf cascade governing nonautonomous control of rib specification within global vertebral domains. Dev. Cell..

[61] Hassan MQ (2009). Molecular switches involving homeodomain proteins, HOXA10 and RUNX2 regulate osteoblastogenesis. Cells Tissues Organs.

[62] Gordon JA (2011). Epigenetic regulation of early osteogenesis and mineralized tissue formation by a HOXA10-PBX1-associated complex. Cells Tissues Organs.

[63] Liao L (2013). Redundant miR-3077-5p and miR-705 mediate the shift of mesenchymal stem cell lineage commitment to adipocyte in osteoporosis bone marrow. Cell Death Dis..

[64] Hu Z (2015). miRNA-132-3p inhibits osteoblast differentiation by targeting Ep300 in simulated microgravity. Sci. Rep..

[65] Hu M (2013). Dynamic fluid flow mechanical stimulation modulates bone marrow mesenchymal stem cells. Bone Res..

[66] Wang H (2018). Osteoblast-targeted delivery of miR-33-5p attenuates osteopenia development induced by mechanical unloading in mice. Cell Death Dis..

[67] Stürmer EK (2006). Standardized bending and breaking test for the normal and osteoporotic metaphyseal tibias of the rat: effect of estradiol, testosterone, and raloxifene. J. Bone Min. Res..

