# Supplementary figures and images for: Bone-targeted lncRNA OGRU alleviates unloading-induced bone loss via miR-320-3p/Hoxa10 axis
Source: Cell Death Dis. 2020 May 19;11(5):382. doi: 10.1038/s41419-020-2574-1 (PMC7237470; doi:10.1038/s41419-020-2574-1)

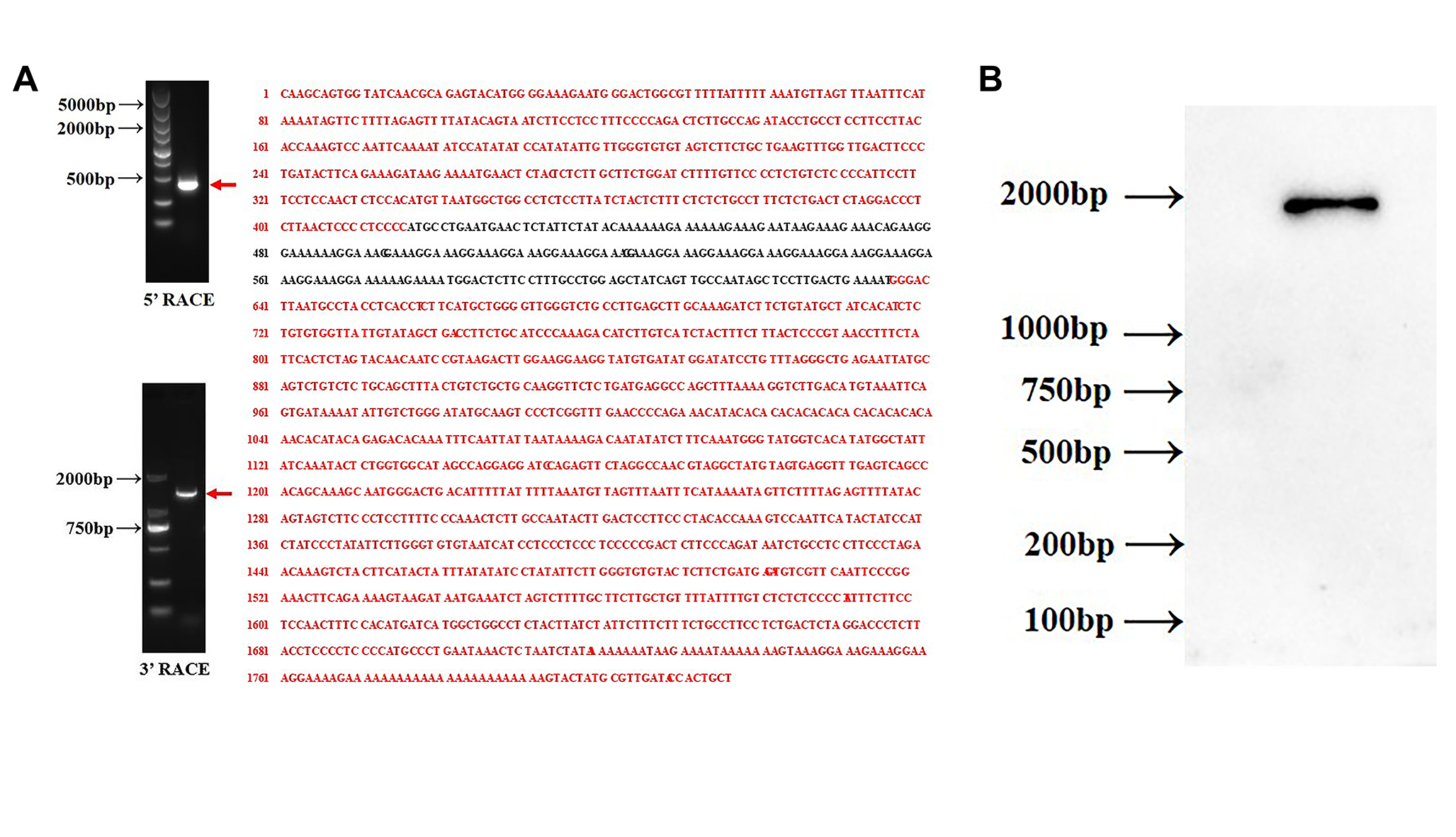

Supplement: Supplementary file 1 — Supplementary Figure 1 [file 41419_2020_2574_MOESM1_ESM.tif]

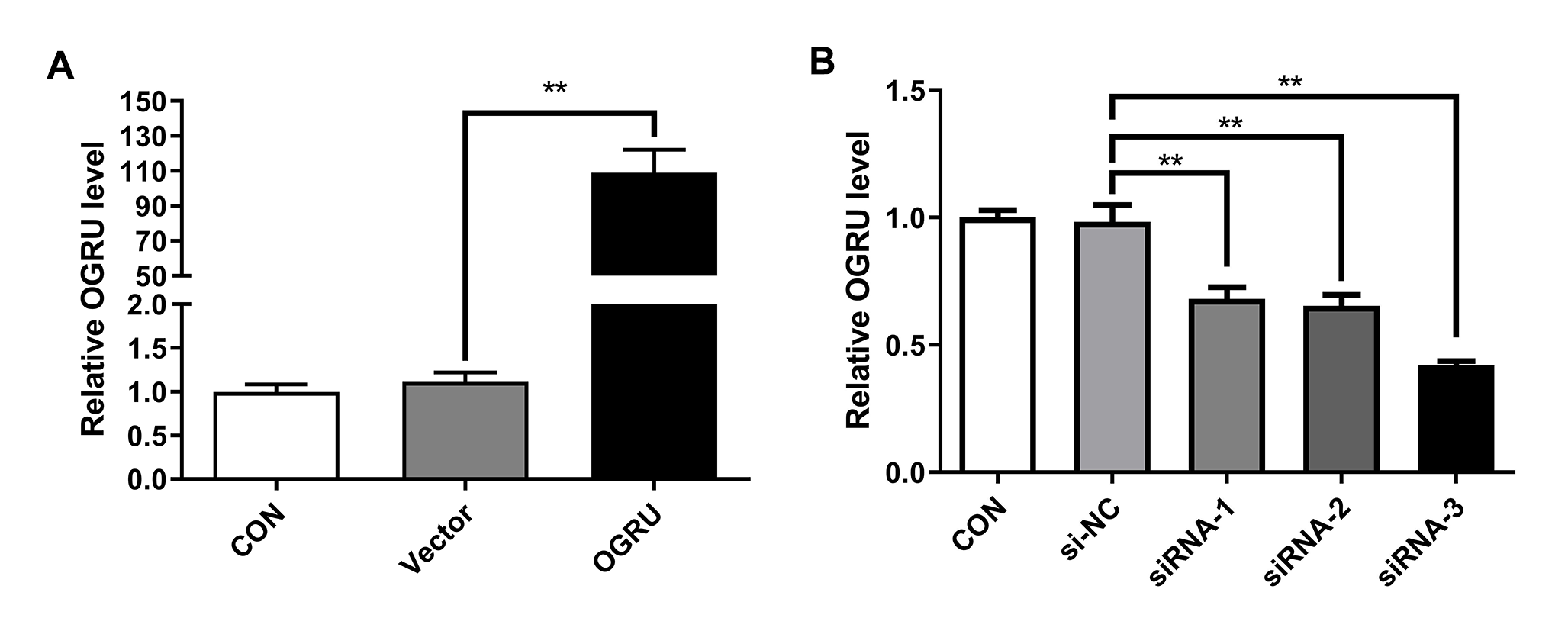

Supplement: Supplementary file 2 — Supplementary Figure 2 [file 41419_2020_2574_MOESM2_ESM.tif]

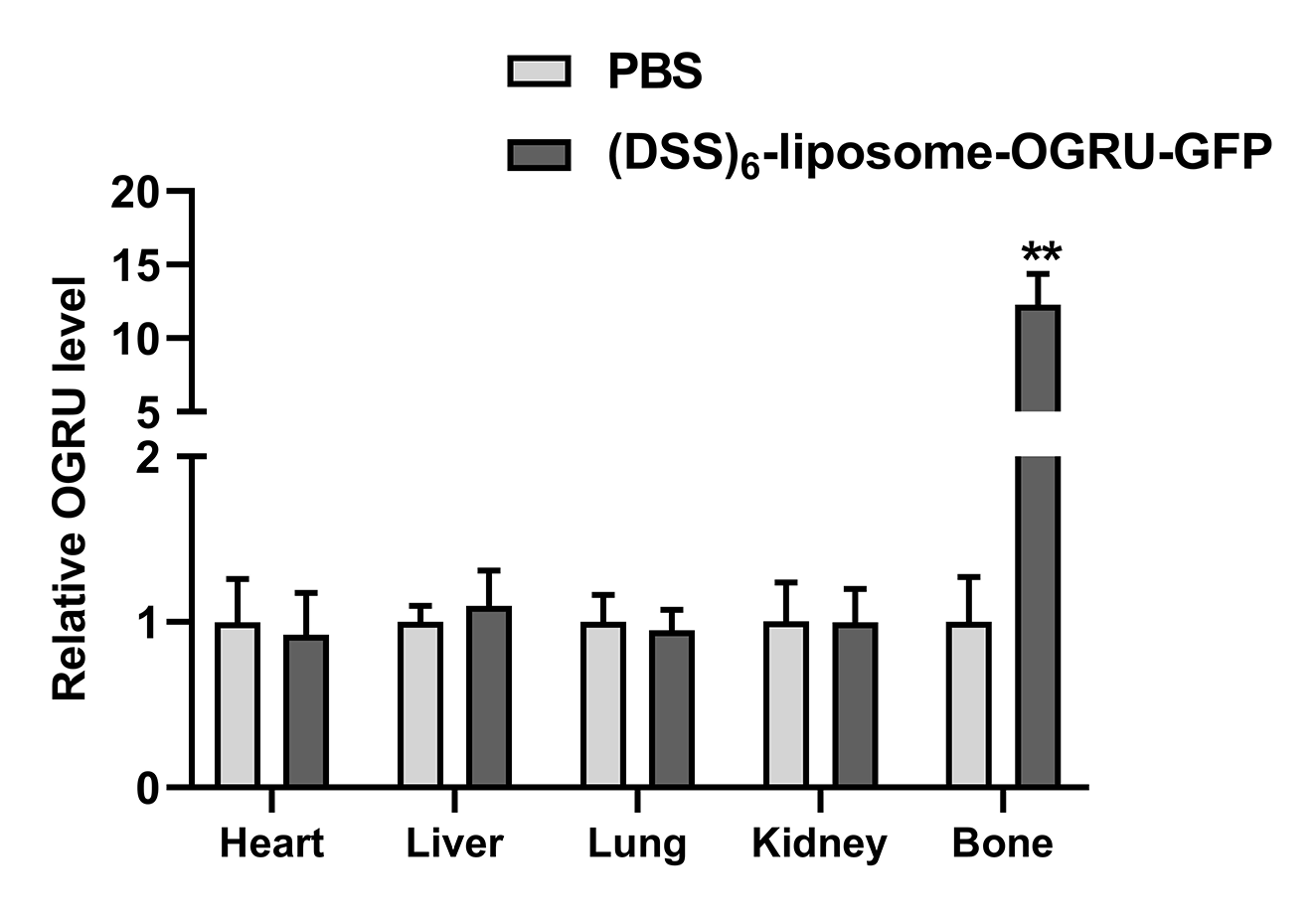

Supplement: Supplementary file 3 — Supplementary Figure 3 [file 41419_2020_2574_MOESM3_ESM.tif]

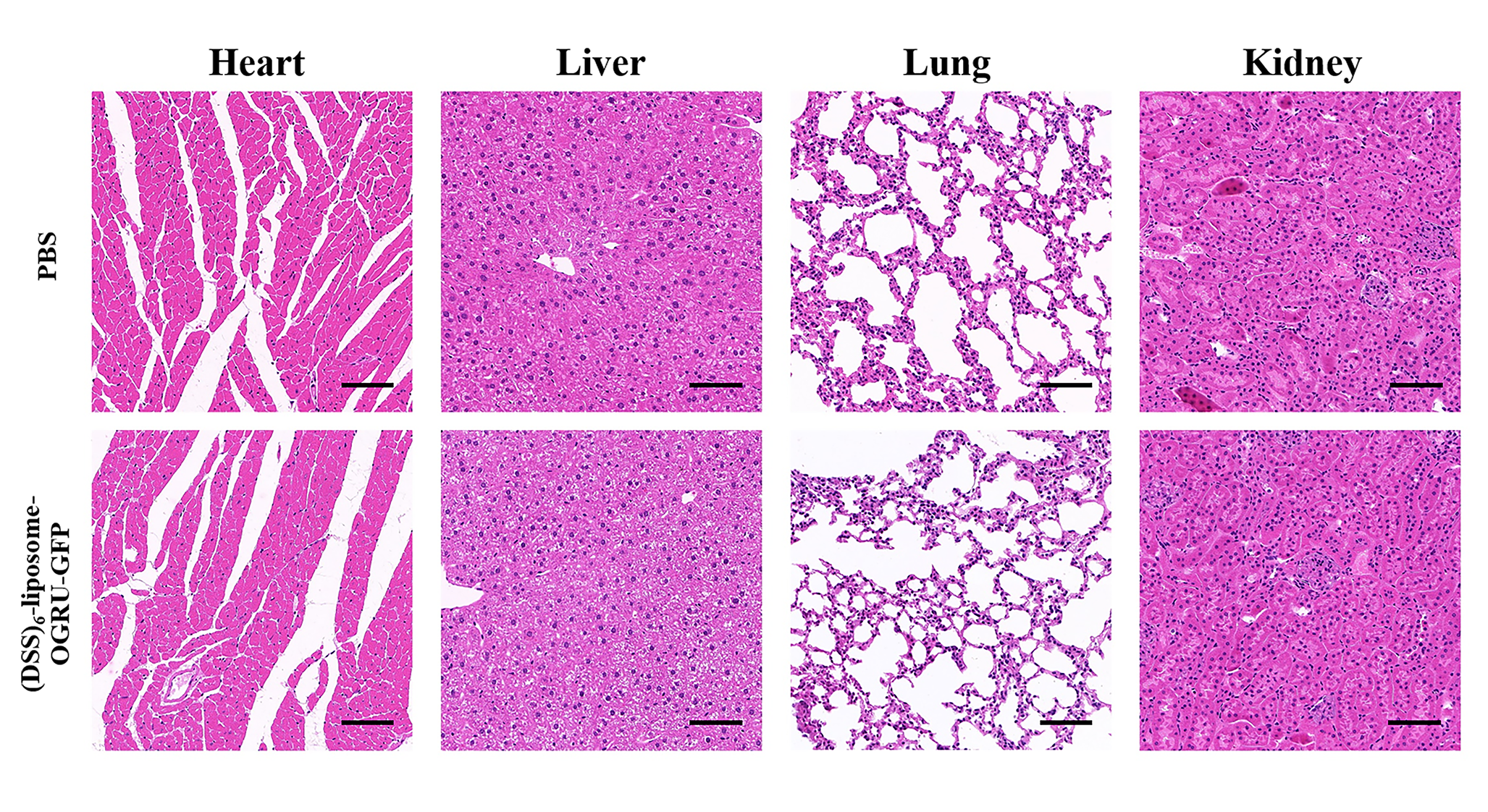

Supplement: Supplementary file 4 — Supplementary Figure 4 [file 41419_2020_2574_MOESM4_ESM.tif]

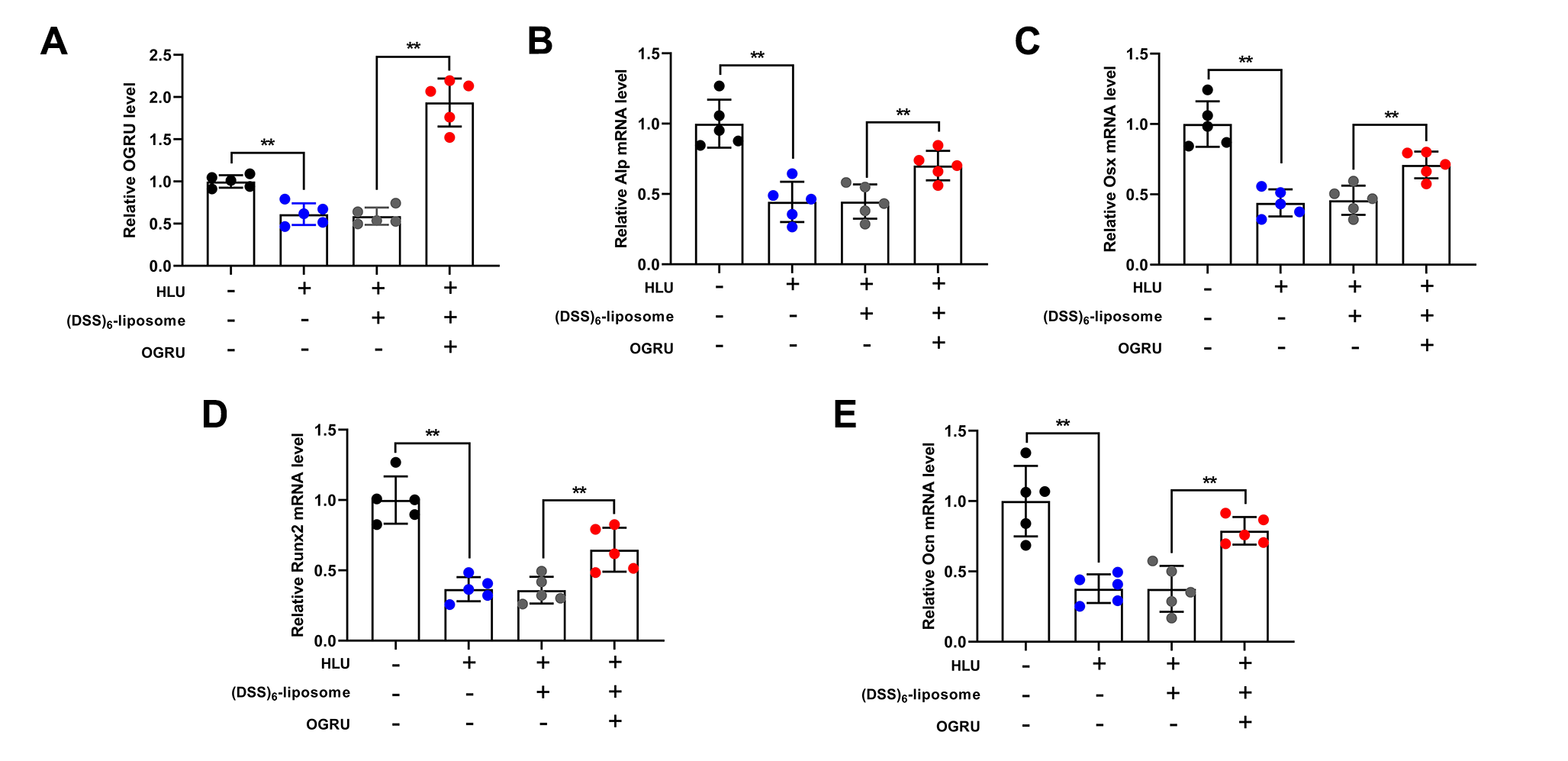

Supplement: Supplementary file 5 — Supplementary Figure 5 [file 41419_2020_2574_MOESM5_ESM.tif]

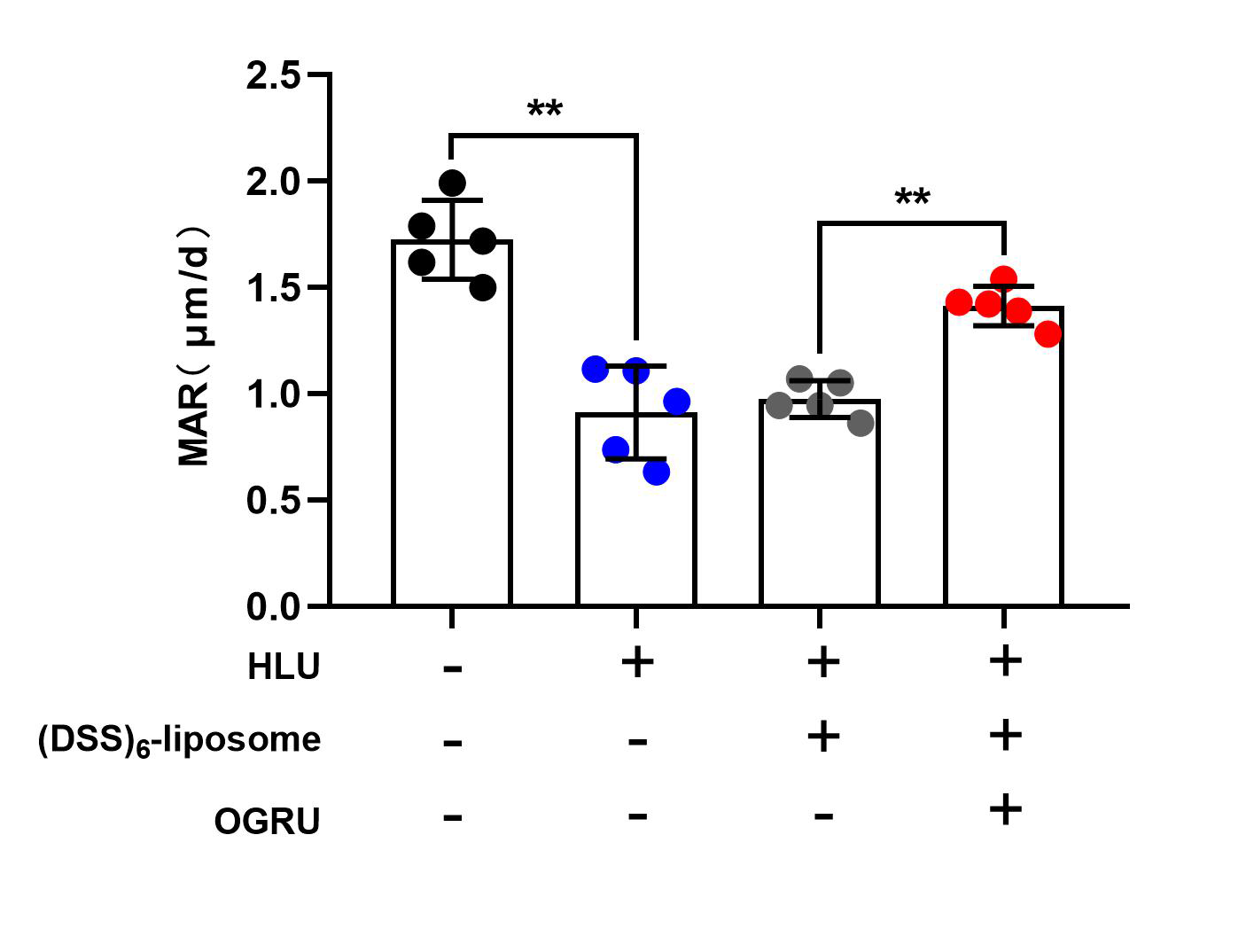

Supplement: Supplementary file 6 — Supplementary Figure 6 [file 41419_2020_2574_MOESM6_ESM.tif]

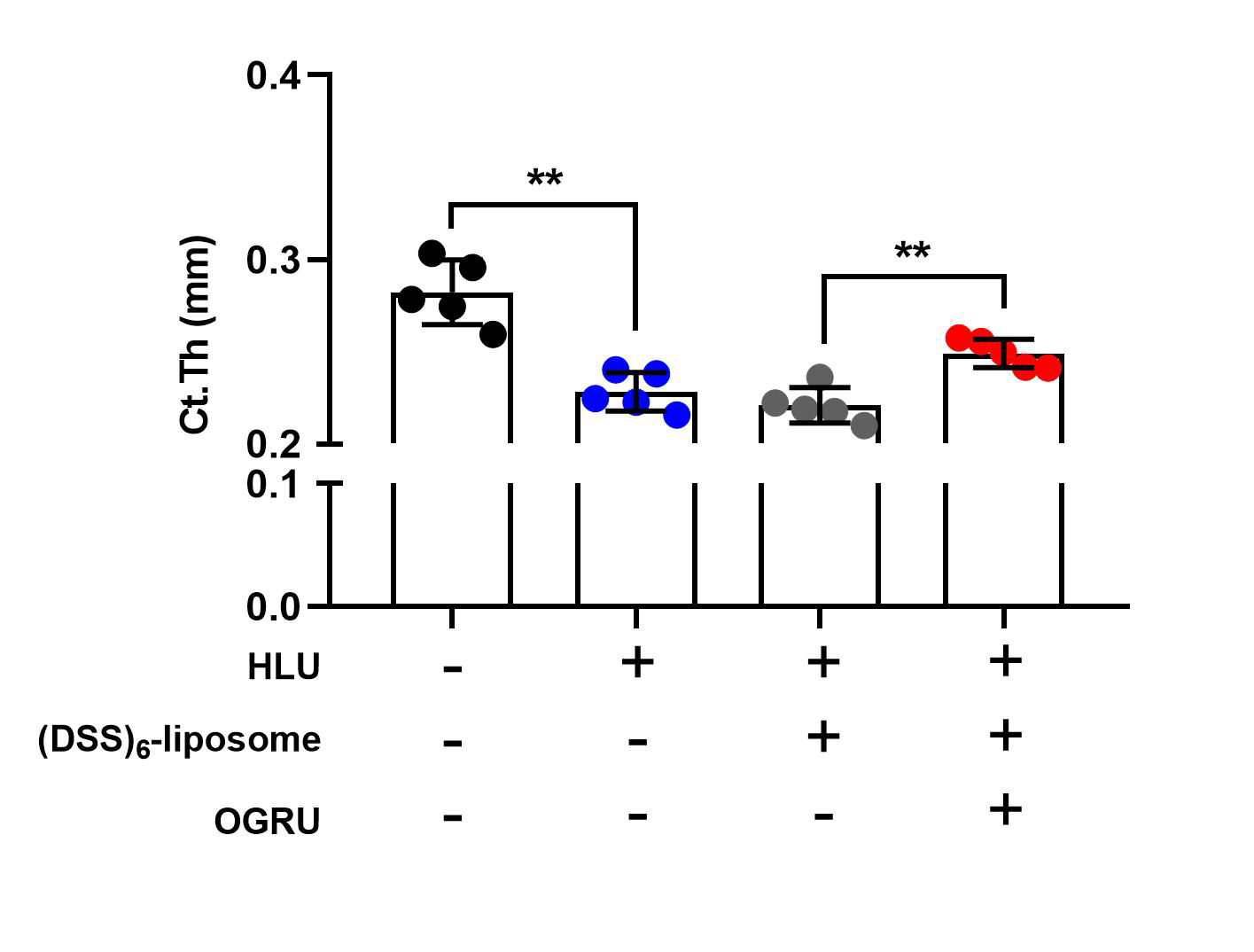

Supplement: Supplementary file 7 — Supplementary Figure 7 [file 41419_2020_2574_MOESM7_ESM.tif]

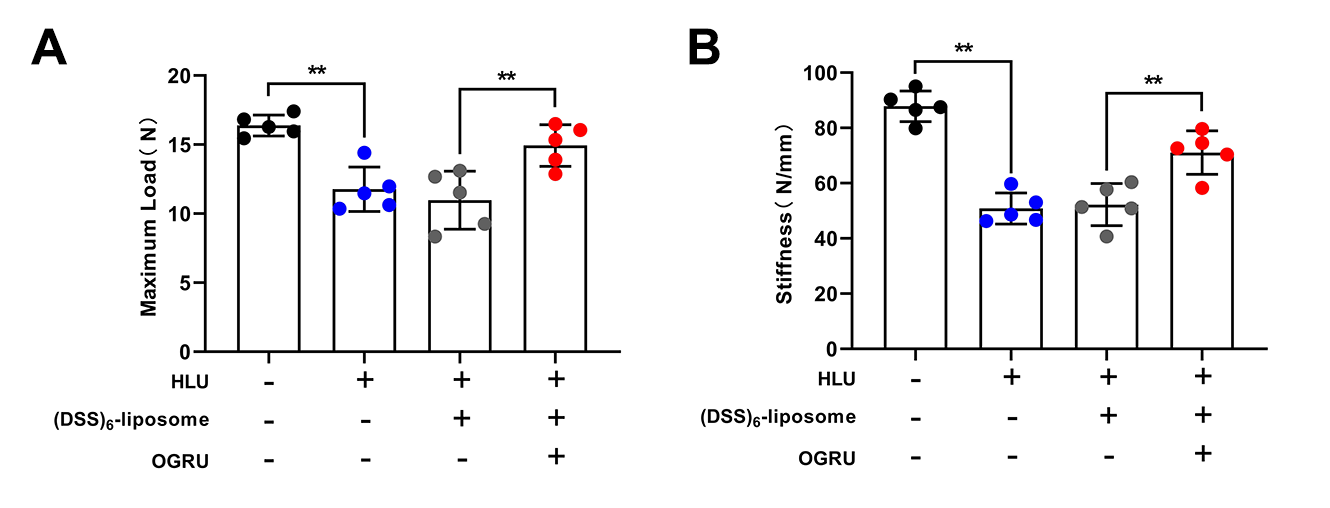

Supplement: Supplementary file 8 — Supplementary Figure 8 [file 41419_2020_2574_MOESM8_ESM.tif]
