# Supplementary material for: Bone-targeted lncRNA OGRU alleviates unloading-induced bone loss via miR-320-3p/Hoxa10 axis
Source: Cell Death Dis. 2020 May 19;11(5):382. doi: 10.1038/s41419-020-2574-1 (PMC7237470; doi:10.1038/s41419-020-2574-1)
Supplement: Supplementary file 9 — Supplemental Figure Legends and Tables [file 41419_2020_2574_MOESM9_ESM.docx]

**Supplementary Materials for**

**Bone-targeted lncRNA OGRU alleviates unloading-induced bone loss via miR-320-3p/Hoxa10 axis**

**Supplementary Figure 1. Nucleotide sequence of the full-length OGRU. (A)** Left: representative images of PCR products from the 5'-RACE and 3'-RACE procedure in MC3T3-E1 cells. Right: nucleotide sequence of the full-length OGRU (n=3). **(B)** Full length of OGRU was confirmed by northern blot analysis in MC3T3-E1 cells (n=3).

**Supplementary Figure 2. The transfection effect of pcDNA3.1(+)-OGRU and si-OGRU.** qRT-PCR analysis of OGRU expression in MC3T3-E1 cells transfected with pcDNA3.1(+)-OGRU **(A)**, si-OGRU **(B)** or the corresponding controls (n=3). All data are the mean ± SD. ***P* <0.01.

**Supplementary Figure 3. The OGRU expression in different tissues.** qRT-PCR was performed to detect OGRU expression in different tissues (heart, liver, lung, kidney and bone) three days after a single injection of (DSS)_6_-liposome-OGRU-GFP or PBS (n=5). All data are the mean ± SD. ***P* <0.01.

**Supplementary Figure 4. Evaluation of toxicity of (DSS)_6_-liposome delivery system in vivo.** Representative images of H&E staining for heart, liver, lung and kidney were shown three days after a single injection of (DSS)_6_-liposome-OGRU-GFP or PBS (n=5). Scale bars = 100 μm.

**Supplementary Figure 5. (A)** qRT-PCR analysis of OGRU expression in the left tibias of mice (n=5). **(B-E)** qRT-PCR detection of the Alp, Osx, Runx2 and Ocn mRNA levels in the left tibias of mice in the indicated groups (n=5). All data are the mean ± SD. ***P* <0.01.

**Supplementary Figure 6. The widths between the two calcein-labeled lines were measured with Image J (n=5).** All data are the mean ± SD. ***P* <0.01.

**Supplementary Figure 7. Micro-CT measurement of the cortical thickness (Ct.Th) (n=5).** All data are the mean ± SD. ***P* <0.01.

**Supplementary Figure 8. The maximum load and stiffness measured by three-point bending test (n=5).** All data are the mean ± SD. ***P* <0.01.

**Table S1. The protein-coding potential predicted by the Coding Potential Calculator (CPC) and the Coding-Potential Assessment Tool (CPAT).**

| **Method** | **OGRU Score** | **Protein-Coding Cutoff** |
| --- | --- | --- |
| **CPAT** | -0.133633 | >0.44 |
| **CPC** | -0.800224 | >0 |

**Table S2. Binding sites for miRNAs contained in OGRU sequence predicted by RegRNA 2.0.**

| **Motif Name** | **Position** | **Length** | **Minimum Free Energy** | **Score** |
| --- | --- | --- | --- | --- |
| mmu-miR-466i-5p | 979~998 | 20 | -38.3 | 190 |
| mmu-miR-1187 | 978~1000 | 23 | -31.6 | 177 |
| mmu-miR-669n | 988~1007 | 20 | -25.7 | 174 |
| mmu-miR-710 | 775~796 | 22 | -29.17 | 172 |
| mmu-miR-1940 | 266~292 | 27 | -29.04 | 172 |
| mmu-miR-1940 | 1545~1569 | 25 | -27.36 | 171 |
| mmu-miR-1249-5p | 1335~1368 | 34 | -34.9 | 168 |
| mmu-miR-1892 | 1249~1270 | 22 | -32.49 | 168 |
| mmu-miR-5113 | 1605~1629 | 25 | -23.92 | 167 |
| mmu-miR-98-5p | 594~619 | 26 | -20.98 | 165 |
| mmu-miR-574-5p | 978~1000 | 23 | -34.2 | 165 |
| mmu-miR-1892 | 74~95 | 22 | -32.06 | 164 |
| mmu-miR-1943-5p | 1338~1362 | 25 | -26.25 | 163 |
| mmu-miR-1249-5p | 267~291 | 25 | -31.8 | 161 |
| mmu-miR-320-3p | 877~901 | 25 | -21.93 | 160 |
| mmu-miR-709 | 93~116 | 24 | -29.51 | 159 |
| mmu-miR-1954 | 840~860 | 21 | -20.47 | 159 |
| mmu-miR-1249-5p | 1544~1568 | 25 | -29.47 | 158 |
| mmu-miR-669f-5p | 984~1007 | 24 | -24.53 | 158 |
| mmu-miR-1906 | 850~876 | 27 | -24.61 | 158 |
| mmu-miR-149-3p | 1348~1366 | 19 | -28.83 | 157 |
| mmu-miR-3103-5p | 1240~1259 | 20 | -30.83 | 157 |
| mmu-let-7e-5p | 594~619 | 26 | -21.84 | 156 |
| mmu-let-7e-5p | 1628~1650 | 23 | -23.17 | 156 |
| mmu-miR-466f | 976~997 | 22 | -23.35 | 156 |
| mmu-miR-709 | 1371~1395 | 25 | -25.58 | 155 |
| mmu-miR-504-3p | 1535~1554 | 20 | -21.47 | 155 |
| mmu-miR-3103-5p | 1343~1363 | 21 | -23.34 | 155 |
| mmu-miR-5113 | 328~352 | 25 | -24.49 | 155 |
| mmu-miR-652-5p | 612~638 | 27 | -30.73 | 154 |
| mmu-miR-3113-5p | 1090~1113 | 24 | -22.42 | 154 |
| mmu-miR-423-5p | 326~348 | 23 | -27.14 | 153 |
| mmu-miR-1249-5p | 94~121 | 28 | -27.49 | 152 |
| mmu-miR-3059-3p | 1383~1406 | 24 | -24.09 | 152 |
| mmu-miR-3059-3p | 546~567 | 22 | -21.47 | 152 |
| mmu-miR-666-3p | 842~861 | 20 | -20.21 | 152 |
| mmu-miR-708-5p | 562~589 | 28 | -20.43 | 152 |
| mmu-miR-3097-3p | 1137~1158 | 22 | -23.97 | 152 |
| mmu-miR-221-3p | 1125~1149 | 25 | -20.75 | 151 |
| mmu-miR-1249-5p | 1267~1295 | 29 | -21.36 | 151 |
| mmu-miR-710 | 1265~1286 | 22 | -21.5 | 151 |
| mmu-miR-5110 | 1374~1398 | 25 | -27.27 | 151 |
| mmu-miR-204-3p | 1362~1383 | 22 | -22.56 | 150 |
| mmu-miR-298-5p | 538~561 | 24 | -23.54 | 150 |
| mmu-miR-326-5p | 1372~1395 | 24 | -26.94 | 150 |
| mmu-miR-504-3p | 1358~1380 | 23 | -24.87 | 150 |
| mmu-miR-3474 | 1363~1384 | 22 | -24.09 | 150 |
| mmu-miR-5110 | 1543~1567 | 25 | -26.46 | 150 |
| mmu-miR-5110 | 1628~1653 | 26 | -32.16 | 150 |

**Table S3. Bioinformatic prediction of binding sites for miR-320-3p contained in Hoxa10 3’UTR sequence.**

**3.1 Prediction of miR-320-3p target genes using TargetScan**

| Gene Symbol | Position | miSVR score |
| --- | --- | --- |
| Hoxa10 | 658-666 | -1.0087 |
| Hoxa10 | 1190-1205 | -0.4180 |

**3.2 Prediction of miR-320-3p target genes using miRanda**

| Gene Symbol | Position | Context++ score percentile |
| --- | --- | --- |
| Hoxa10 | 660-666 | 96 |
| Hoxa10 | 1200-1206 | 83 |

**3.3 Prediction of miR-320-3p target genes using miRDB**

| Gene Symbol | Position | Target Score |
| --- | --- | --- |
| Hoxa10 | 660-666; 1200-1206 | 81 |

**Table S4. Primers used for qRT-PCR**

| **Gene symbol** | **Forward 5' - 3'** | **Reverse 5' - 3'** |
| --- | --- | --- |
| **GAPDH** | TGTGTCCGTCGTGGATCTGA | TTGCTGTTGAAGTCGCAGGAG |
| **NONMMUT016667** | GCCCTGGCCTGGAACTTACT | AGAAAGCTGGGTGTGGTGGA |
| **NONMMUT006040** | GGTACAACACAGCCGATACACCTG | CAGCGTCCATTCCTTCTCACACTC |
| **NONMMUT040235** | CATTCGCTGAGTACGCTGCTCTG | TACCAGTAGTAGCCTGCCAGCAC |
| **OGRU** | CCGTAAGACTTGGAAGGAAGGTATGTG | CATCAGAGAACCTTGCAGCAGACAG |
| **NONMMUT017108** | AGCATGTGTCCTTCTTACCAGTTGG | GCAGATGCTGGCGAGGATGTG |
| **NONMMUT056478** | GGGTATGACTCAGTGACAAGGA | CTGTCATGGTATCTGGCCCAA |
| **Osx** | AAGAGGTTCACCCGCTCTGA | TGATGTTTGCTCAAGTGGTCG |
| **Runx2** | GAACCAAGAAGGCACAGACAGA | GGCGGGACACCTACTCTCATAC |
| **Alp** | GCAGTATGAATTGAATCGGAACAAC | ATGGCCTGGTCCATCTCCAC |
| **Ocn** | GACCGCCTACAAACGCATCTA | CAGAGAGAGAGGACAGGGAGGA |
| **12s rRNA** | TCGATAAACCCCGCTCTACCT | TGGCTACACCTTGACCTAACGTT |
| **45s rRNA** | GTGCCCTCACGTGTTTCACTTT | TAGGAGACAAACCTGGAACGCT |
| **Hoxa10** | TTCGCCGGAGAAGGACTC | TCTTTGCTGTGAGCCAGTTG |
| **U6** | CTCGCTTCGGCAGCACA | AACGCTTCACGAATTTGCGT |
| **mmu-miR-98-5p** | GCTGAGGTAGTAAGTTGTATTG | The 3' primer for qRT-PCR analysis of miRNAs expression is the mRQ 3' Primer supplied with the Mir-X™ miRNA First-Strand Synthesis kit (Clontech, USA). |
| **mmu-miR-320-3p** | AAAAGCTGGGTTGAGAGGGC |  |
| **mmu-miR-221-3p** | CTACATTGTCTGCTGGGTTTC |  |
| **mmu-miR-708-5p** | GAGCTTACAATCTAGCTGGG |  |

**Table S5. RACE primers for OGRU**

| **Gene specific primer** | **Sequence 5' - 3'** |
| --- | --- |
| **3' RACE-outer** | AGACTCTTGCCAGATACCTGCCTCC |
| **3' RACE-inner** | GGGACTTAATGCCTACCTCACCT |
| **5' RACE-outer** | AGAGTTTATTCAGGGCATGGGGAGG |
| **5' RACE-inner** | GGGGAGGGGAGTTAAGAGGGTCC |

**Table S6. siRNAs sequences**

| **Name** | **Sequence 5' - 3'** |
| --- | --- |
| **siRNA-OGRU sense** | GCUGGCCUCUACUUAUCUATT |
| **siRNA-OGRU antisense** | UAGAUAAGUAGAGGCCAGCTT |
| **siRNA-Hoxa10 sense** | CCAACUGGCUCACAGCAAATT |
| **siRNA-Hoxa10 antisense** | UUUGCUGUGAGCCAGUUGGTT |
| **siRNA-control sense** | UUCUCCGAACGUGUCACGUTT |
| **siRNA-control antisense** | ACGUGACACGUUCGGAGAATT |
